# Fabrication of Organic/Inorganic Nanocomposites: From Traditional Synthesis to Additive Manufacturing

**DOI:** 10.1002/adma.202505504

**Published:** 2025-06-23

**Authors:** Liwen Zhang, Xumin Huang, Liwei Liu, Naufal Kabir Ahamed Nasar, Xinyan Gu, Thomas P. Davis, Xiaoyu (Rayne) Zheng, Lianzhou Wang, Ruirui Qiao

**Affiliations:** ^1^ Australian Institute for Bioengineering and Nanotechnology The University of Queensland Brisbane QLD 4072 Australia; ^2^ Department of Material Science and Engineering University of California Berkeley CA 94720 USA

**Keywords:** 3D printing, biomedicine, hybrid nanoparticles, soft robots, water treatment

## Abstract

Nanocomposites, are materials that incorporate nanosized particles into a matrix of standard material, have emerged as a versatile class of materials with tunable properties for a wide range of applications. Traditional fabrication approaches, including physical blending, in situ polymerization, layer‐by‐layer assembly, and sol–gel synthetic methods, have been widely employed to develop nanocomposites with high structural homogeneity and tailored properties. This review presents a cohesive and comprehensive overview of nanocomposite fabrication methods, spanning from conventional synthetic strategies to cutting‐edge approaches such as 3D printing technologies. How 3D printing has driven innovations in nanocomposite applications, particularly in biomedicine, soft robotics, electronics, and water treatment, is explored. Additionally, key challenges in 3D‐printed nanocomposite development are discussed, and emerging advancements such as 5D printing, artificial intelligence (AI)‐assisted material optimization, nanoscale additive manufacturing, and closed‐loop recycling systems are highlighted. By bridging traditional synthesis with cutting‐edge fabrication techniques, this review aims to provide insights into the future directions of nanocomposite research and applications.

## Graphical abstract

It provides an overview of the synthesis of organic/inorganic nanocomposites, spanning from conventional methods to advanced 3D printing techniques. It summarizes key challenges in 3D‐printed nanocomposites and highlights emerging innovations, including 5D printing, AI‐assisted material design, nanoscale additive manufacturing, and sustainable closed‐loop recycling systems.

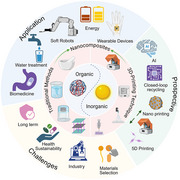

## 1 Introduction

Hybrid nanoparticles in which inorganic components (such as metal and metalloid particles, their oxides, and more) and organic components are intricately interconnected, resulting in advanced materials with tailored properties. Hybrid nanoparticles consist of two or more distinct nanoparticles integrated into a single functional structure that remains within the nanoscale range.^[^ ^1^ ^]^ For example, inorganic nanoparticles—including gold nanoparticles (AuNPs), iron oxide nanoparticles (IONPs), quantum dots (QDs), carbon dots, and liquid metal nanoparticles (LMNPs)—can be functionalized with organic molecules such as small molecules, polymers, and biomolecules. This functionalization enables the formation of hybrid nanoparticles with enhanced or novel properties, allowing for a broad range of applications spanning optics, electronics, energy, environmental science, drug delivery, biomedicine, and tissue engineering.

**Figure 1:**
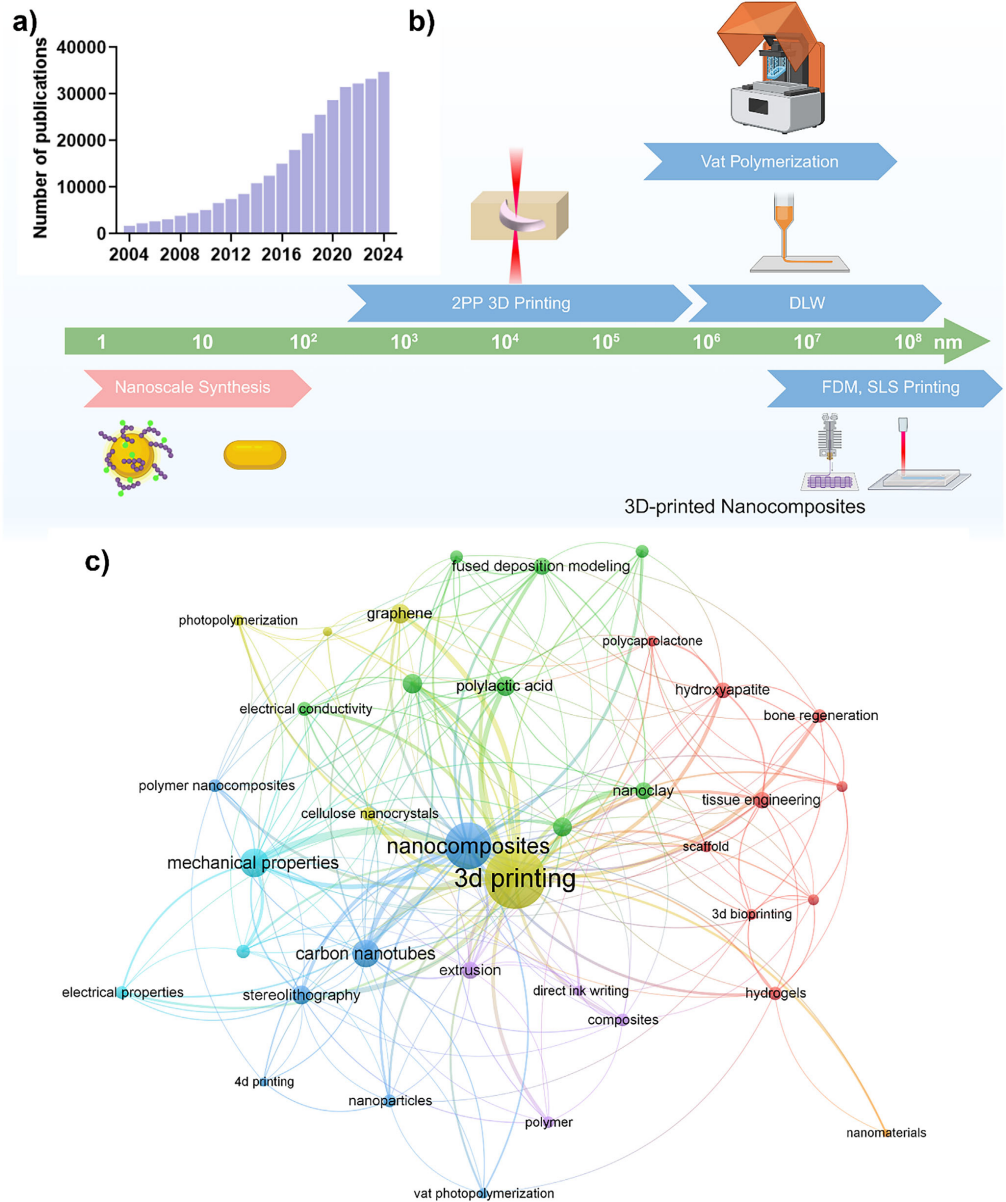
a) Number of publications (2004–2024) retrieved from Web of Science using the keywords “(Nanocomposites* OR nanoparticle* OR nanomaterial*) AND (“3D Printing” OR “Additive Manufacturing”)”; b) Size range spanning nanoparticles, hybrid nanoparticles, and nanocomposites fabricated via conventional synthesis or advanced 3D printing techniques; c) Keyword co‐occurrence analysis (2014–2024) visualized using VOSviewer, highlighting dominant terms such as nanocomposites, 3D printing, and mechanical properties, underscoring their research significance.

In recent years, the definition of nanomaterials has broadened to nanocomposite materials, which are formed by dispersing inorganic nanoparticles within a macroscopic organic matrix, provided that at least one of the constituent phases retains dimensions at the nanoscale.^[^ ^2^ ^]^ To achieve well‐defined nanocomposites at the micron to bulk scale, traditional methods such as physical blending and in situ deposition have been employed to fabricate materials with high homogeneity. More recently, additive manufacturing (3D printing) has emerged as a powerful tool for fabricating composite materials with exceptional properties and complex structures to meet practical demands, broadening their applications (**Figure** **1**). Compared to traditional methods, 3D printing offers several key advantages, including the ability to fabricate complex architectures across a broad size range, from the micron scale to bulk structures; high customization potential; and improved cost‐effectiveness, environmental sustainability, and ease of operation over conventional manufacturing processes.

This review provides a comprehensive overview of the fabrication of nanocomposites using both traditional and 3D printing methods, focusing on 3D printing as an advanced approach for nanocomposite development. There has been an over‐20‐fold increase in the number of publications, highlighting the rapid development of this field, as shown in Figure 1a. We first summarize the synthesis of nanocomposites (size ranging from nano to microscale), including surface functionalization strategies to enhance their properties (Figure 1b). Next, we introduce state‐of‐the‐art 3D printing techniques for nanocomposite fabrication, spanning from the microscale to bulk structures, and summarize their properties and performance. We examine how these methods have driven innovations across various applications, including biomedicine, soft robotics, electronics, and water treatment. Co‐occurrence analysis highlights highly interdisciplinary research interests, particularly in materials, fabrication approaches, and biomedical applications (Figure 1c). Based on this analysis, we discuss key challenges and future perspectives, highlighting emerging advancements such as 5D printing (3D + time + information), artificial intelligence (AI)‐assisted material optimization, nanoscale additive manufacturing, and closed‐loop recycling systems that could further propel the development of 3D‐printed hybrid materials.

## 2 Synthesis of Nanomaterials

Nanoparticles are with at least one dimension less than 100 nm, and they serve as fundamental building blocks for a wide range of advanced materials due to their unique characteristics, including pronounced size effects, high surface area, and quantum phenomena.^[^ ^3^ ^]^ Building upon the functional versatility of nanomaterials, hybrid nanoparticles integrate distinct components—typically combining organic and inorganic constituents—at the nanoscale to synergistically enhance performance.^[^ ^4^ ^]^ By carefully designing the interface between different phases, hybrid nanoparticles can exhibit tailored optical, electrical, or catalytic properties that exceed those of the individual components, enabling multifunctionality within a single material platform.^[^ ^5^ ^]^ Various inorganic nanoparticles (e.g., AuNPs, IONPs, QDs, LMNPs) and organic compounds (e.g., small molecules, polymers, biomolecules, and dendrimers) are widely utilized to fabricate hybrid nanoparticles.^[^ ^6^ ^]^ To the best of our knowledge, hybrid nanoparticles have demonstrated pivotal roles in a wide range of biomedical applications, including imaging, drug delivery, phototherapy, and theranostics.^[^ ^7^ ^]^ In the past decades, hybrid nanoparticles have also unveiled their great potential as building blocks for composite materials, including 3D‐printed nanocomposites,^[^ ^8^ ^]^ bioactive scaffolds for tissue engineering,^[^ ^9^ ^]^ conductive and flexible electronics.^[^ ^10^ ^]^ Thus, nanocomposites based on hybrid nanoparticles are fabricated as multiphase materials in which one phase has at least one nanoscale dimension and is dispersed within a matrix of another material, typically polymeric, ceramic, or metallic.^[^ ^11^ ^]^ The incorporation of hybrid nanoparticles as fillers or reinforcements improves mechanical strength, thermal stability, electrical conductivity, or other performance metrics of nanocomposites.

**Figure 2:**
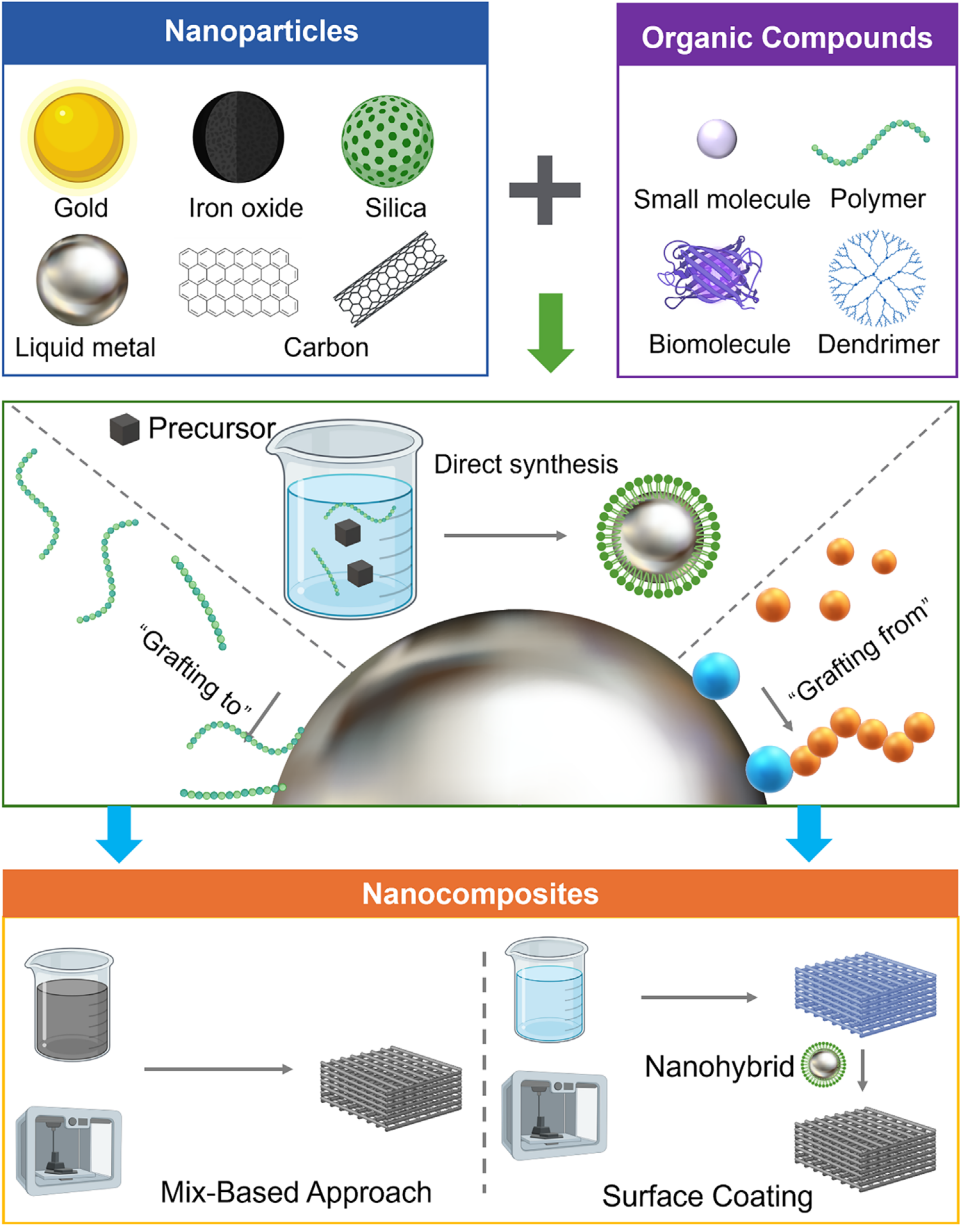
Overview of synthesis strategies of nanomaterials ranging from nanoparticles and hybrid nanoparticles to nanocomposites. Created with BioRender.com.

This section examines the synthetic strategies for nanoparticles and hybrid nanoparticles, with a focus on achieving precise interface control, improved uniformity, and enhanced reproducibility at the nanoscale‐factors that are critical for enabling simple and efficient fabrication at the macroscale (**Figure** **2**). Building upon these well‐established nanoscale building blocks, we then present a detailed comparison between traditional and emerging approaches for fabricating nanocomposites. Furthermore, we systematically illustrate how the incorporation of hybrid nanoparticles influences the structural and functional performance of the resulting nanocomposites.

### 2.1 Synthetic Approaches of Nanoparticles

In the mid‐20th century, various advanced fabrication techniques for the synthesis of nanoparticles have continuously emerged, opening a new era of nanotechnology. These approaches are broadly classified into top‐down and bottom‐up methodologies, as summarized in **Table** **1**, Entry 1–4.^[^ ^12^ ^]^ In top‐down strategies, mechanical techniques, like ball milling, are considered some of the most cost‐effective and straightforward approaches for bulk nanoparticle production.^[^ ^13^ ^]^ Ball milling operates through mechanical attrition, wherein kinetic energy from a grinding medium is transferred to bulk materials, leading to size reduction without the need for complex chemical processing. Thermal methods (e.g., electrospinning) combine heat and electric fields to produce nanoparticles from polymer solutions, offering a versatile platform for generating fibrous nanostructures and oxide‐based nanoparticles.^[^ ^14^ ^]^ Additionally, high‐energy techniques (e.g., arc discharge,^[^ ^15^ ^]^ laser ablation,^[^ ^16^ ^]^ solar flux,^[^ ^17^ ^]^ and plasma‐assisted synthesis)^[^ ^18^ ^]^ utilize excessive energy input in the form of heat, electricity, or light to initiate nanoparticle formation. For example, arc discharge is one of the earliest controlled techniques for synthesizing carbon nanotubes (CNTs), utilizing a high‐voltage electrical discharge between graphite electrodes to vaporize carbon and facilitate the formation of nanotube structures.^[^ ^19^ ^]^ Despite their effectiveness, these techniques are often constrained by high energy consumption and limited scalability. Among these high‐energy techniques, solar flux approaches are particularly attractive for industrial‐scale applications due to the renewable and abundant nature of sunlight. Top‐down chemical methods, such as anodizing, involve electrochemical processes that etch or oxidize bulk materials into nanoscale features.^[^ ^20^ ^]^ While these techniques are easily scalable and widely adopted in industrial settings, they present challenges related to chemical waste generation and environmental impact. Furthermore, lithographic techniques, originally developed for the semiconductor industry, have been adapted for nanostructure fabrication.^[^ ^21^ ^]^ These methods allow for high‐resolution patterning and the formation of ordered nanostructure arrays, but they are limited by the need for specialized equipment and complex operational requirements. These top‐down fabrication techniques provide versatile and scalable strategies for nanoparticle synthesis, each presenting unique strengths and inherent limitations. Ongoing advancements in these methods are essential to meet the evolving demands of precision, efficiency, and environmental sustainability in nanoparticle engineering.

**Table 1:** Synthesis of nanomaterials: approaches, advantages, and drawbacks.

Entry	Methods	Fabrication of nanomaterials	Advantages	Drawbacks	Refs.
1	Ball milling	Mechanical attrition using grinding media to reduce bulk materials into nanoparticles	Cost‐effective, straightforward, no need for complex chemical processing	Limited control over shape and size, high defect densities	[59]
2	Electrospinning	Uses electric fields and heat to draw polymer solutions into nanoparticles or oxide nanoparticles	Versatile, high surface area	Post‐processing challenges, limited to specific polymers	[60]
3	High‐energy techniques	High‐voltage discharge between electrodes to form nanoparticles	High purity	High energy consumption, limited scalability	[61]
4	Vapor‐phase methods	Deposition of thin films via CVD, PVD, or ALD onto nanocomposites	High purity, conformal coatings, precise control at nanoscale	Requires vacuum systems	[27]
5	In situ synthesis	In situ nucleation of nanoparticles in the presence of organic components	Simple synthesis, minimized purification steps	Less functionalization, some side reactions	[7]
6	“Grafting to” approach	Polymer chemically attached to the nanoparticle surface	Well‐defined polymer structures, uniform surface coating, mild reaction	Time‐consuming, low grafting density caused by steric hindrance	[7]
7	“Grafting from” approach	Initiators attached to the nanoparticle surface, followed by subsequent polymerization	High grafting density, strong interaction between nanoparticles and polymers	Complex synthesis procedures, less control over polymer structures	[7]
8	Melt blending	Mixing nanoparticles with polymers in a molten state	Cost‐effective, scalable, and Solvent‐free	Nanoparticle aggregation, high temperature, and limited to thermally sensitive nanoparticles	[52]
9	Solution blending	Dispersing nanoparticles in a polymer solution	Simple process, good dispersion, scalable production	Potential toxicity, phase separation issues	[52]
10	Sol–gel method	Hydrolysis and polycondensation	Precise control, high purity, low temperature	Brittle final product requires aging and drying	[54b]
11	In situ polymerization	Dispersing nanoparticles in monomer	Good dispersion, strong interfacial bonding, and tunable properties	Long reaction time, the use of specific initiators	[53a]
12	Self‐assembly technology	Nanoparticles into structures	Spontaneous organization, high nanoscale, no need for external forces	Limited to materials selection, complex control	[62]
13	Layer‐by‐layer technology	Layer‐by‐layer deposition	Excellent uniformity, precise layer control, and high flexibility in material selection	Time‐consuming, weak stability	[63]

In contrast, bottom‐up approaches build nanostructures from molecular or atomic units, enabling precise control over composition, morphology, and functional properties. Vapor‐phase approaches—including chemical vapor deposition (CVD),^[^ ^22^ ^]^ physical vapor deposition (PVD),^[^ ^23^ ^]^ and atomic layer deposition (ALD)^[^ ^24^ ^]^—enable deposition of nanoparticles with exceptional purity, uniformity, and nanoscale control. In CVD, gaseous precursors react or decompose on a heated substrate to form solid materials, and this method is widely used for growing CNTs and graphene directly onto substrates, facilitating their uniform dispersion within polymer matrices.^[^ ^25^ ^]^ PVD, including sputtering and thermal evaporation, enables the precise coating with thin metal or ceramic films, enhancing their electrical, barrier, and corrosion‐resistant properties without compromising bulk material performance.^[^ ^26^ ^]^ ALD, distinguished by its atomic‐level control, is especially effective for applying conformal coatings and tuning interfacial interactions in nanomaterials. These vapor‐based techniques have become indispensable for fabricating flexible electronics, energy storage devices, sensors, and high‐performance coatings.^[^ ^27^ ^]^ These bottom‐up approaches offer unparalleled control over the composition and architecture of nanomaterials, making them essential for the future development of nanocomposites. However, challenges related to scalability, structural complexity, and process efficiency must be addressed to fully realize their industrial potential.

### 2.2 Synthetic Approaches of Hybrid Nanoparticles

#### 2.2.1 Direct Synthesis Method

Direct synthesis, as known as “one‐pot” synthesis or “In situ” synthesis, provides a straightforward and efficient approach to fabricating hybrid nanoparticles (Figure 2, Entry 5 in Table 1). Inorganic nanoparticles are directly synthesized in a one‐step reaction in the presence of organic components, which serve as stabilizers to prevent aggregation and structure‐directing agents to control the size, morphologies, and scalability of hybrid nanoparticles.^[^ ^5^ ^]^ In some cases, the organic components also are regarded as reducing agents to participate in the formation reaction of hybrid nanoparticles. Du and colleagues employed a one‐pot synthesis method to fabricate hybrid nanoparticles composed of polyethyleneimine (PEI) and graphene. In this process, PEI functioned as a reducing agent, enabling the efficient reduction of graphene oxide (GO) while simultaneously enhancing the dispersion and stability of the resulting hybrid nanoparticles.^[^ ^28^ ^]^ The organic components also play a crucial role in regulating the nucleation, growth, and assembly of inorganic nanoparticles, allowing for precise control over the size and shape of hybrid nanoparticles. Gao and co‐workers reported the “one‐pot” synthesis of Fe_3_O_4_‐based hybrid nanoparticles by coating poly(ethylene glycol) (PEG) as a stabilizer and using ferric triacetylacetonate as precursors.^[^ ^29^ ^]^ In comparison with conventional aqueous methods, the resulting hybrid nanoparticles exhibit a significantly narrower size distribution and precise size control within the range of 4 to 18 nm. This enhanced uniformity is attributed to the unique molecular interactions between Fe(acac)₃ and PEG, which regulate nucleation and growth dynamics. Furthermore, direct synthesis enables precise morphological control of hybrid nanoparticles through self‐assembly. Yang et al. explored a series of polystyrene‐*block*‐poly(4‐vinylpyridine) copolymers to direct the self‐assembly of gold‐based hybrid nanoparticles with various morphologies ranging from dumbbell‐like to multilobed, flower‐like architectures.^[^ ^30^ ^]^

However, achieving uniform dispersion and scalable fabrication of hybrid nanoparticles remain critical challenges. To address this, Davis and co‐workers synthesized three reversible addition‐fragmentation chain‐transfer polymerization (RAFT) polymers respectively containing phosphonic acids, carboxylic acids, and glycerol groups as anchoring groups for the co‐precipitation synthesis of IONPs,^[^ ^31^ ^]^ which revealed that phosphonic acid‐based polymers have the highest grafting density and provide best colloidal stability.^[^ ^32^ ^]^ This finding highlights the importance of selecting appropriate polymer anchoring groups in direct synthesis strategies to significantly enhance the dispersibility and stability of hybrid nanoparticles. To address the challenges of scale‐up, Yu and co‐workers proposed a scalable one‐pot synthesis method for the synthesis of silica nanoparticles at the hundred‐gram scale.^[^ ^33^ ^]^ Developing scalable and eco‐friendly synthesis approaches will be crucial for expanding the practical applications of hybrid nanoparticles in biomedicine, catalysis, and advanced manufacturing. In addition, sol–gel processing has garnered considerable attention for the synthesis of hybrid nanoparticles due to its ability to merge the structural rigidity of ceramics with the functional versatility of polymers.^[^ ^34^ ^]^ The sol–gel process involves the hydrolysis and condensation of metal alkoxide precursors in the presence of polymeric or nanoscale components, leading to the formation of a homogeneously integrated hybrid network at the nanoscale.^[^ ^35^ ^]^ These hybrid nanoparticles exhibit a synergistic combination of properties, including thermal stability, optical clarity, and chemical tunability. A notable example is Ormocers (organically modified ceramics), which are produced by introducing polymerizable organic groups into an inorganic sol–gel matrix. Ormocers are widely recognized for their excellent biocompatibility, wear resistance, and aesthetic appeal, particularly in dental applications such as restorative materials, coatings, and adhesives.^[^ ^2^ ^]^ Sol–gel‐derived materials are increasingly utilized in biomedical devices, protective coatings, and optoelectronics, reflecting their broad applicability and technological promise.

#### 2.2.2 “Grafting to” Strategy

Surface modification of inorganic nanoparticles is a widely utilized strategy in the “grafting to” approach for fabricating hybrid nanoparticles, where polymer chains are covalently attached to the nanoparticle surface via a single chain end (Figure 2, Entry 6 in Table 1). Since the polymers are pre‐synthesized, their molecular weights and polydispersity index are well‐defined prior to surface modification, ensuring controlled surface interactions and uniform surface coating. The well‐defined polymers also enable precise control over grafting density and coating thickness, thereby ensuring enhanced stability, functionality, and compatibility of the hybrid nanoparticles. In the “grafting to” approach, pre‐synthesized polymers are conjugated onto the surface of inorganic nanoparticles in organic solvents (e.g., tetrahydrofuran (THF), dimethylformamide (DMF)) or water (for hydrophilic systems) through mild and efficient coupling reactions. These reactions include 1‐ethyl‐3‐(3‐dimethylaminopropyl) carbodiimide/N‐hydroxysuccinimide (EDC/NHS)‐mediated carboxyl‐amine coupling, dicyclohexylcarbodiimide (DCC)‐catalyzed ester formation, Cu(I)‐catalyzed click chemistry, and the formation of coordination bonds.^[^ ^36^ ^]^ Some inorganic nanoparticles, including QDs,^[^ ^37^ ^]^ GO,^[^ ^38^ ^]^ MoS_2_ nanoflakes,^[^ ^39^ ^]^ upconversion nanoparticles (UCNPs),^[^ ^40^ ^]^ and Fe_3_O_4_ nanoparticles,^[^ ^41^ ^]^ have been successfully conjugated with amino‐terminated PEG using the “grafting to” method to fabricate hybrid nanoparticles. For instance, our group synthesized a series of perfluoropolyether (PFPE)‐terminated polymers with varying self‐assembled folding topologies and physicochemical properties for the surface modification of exceedingly small IONPs.^[^ ^42^ ^]^ Gel permeation chromatography displayed that the PFPE‐terminated polymers were precisely designed with molecular weights ranging from 8000 to 10,000 g/mol and polydispersity indexes below 1.20, achieving a high polymer grafting density of ≈82%.

However, the “grafting to” approach is often limited by a time‐consuming surface coating process, particularly for nanoparticles synthesized in nonpolar solvents. Strategies for transferring nanoparticles from nonpolar, hydrophobic solvents to polar, hydrophilic aqueous environments remain limited, and it is very difficult to completely remove original surface ligands in “grafting to” methods. To address these issues, Murray and co‐workers developed a generalized ligand‐exchange strategy, which provides rapid and efficient phase transfer of nanoparticles.^[^ ^43^ ^]^ Specifically, Nitrosonium tetrafluoroborate (NOBF₄) or diazonium tetrafluoroborate were employed to rapidly replace the original ligands, making nanoparticles homogenous distribution within 10 min in various polar, hydrophilic media such as DMF, dimethyl sulfoxide (DMSO), or acetonitrile. Afterward, the weak binding affinity of BF₄⁻ anions further enabled fully reversible phase transfer of nanoparticles between hydrophobic and hydrophilic media. This generalized strategy has been widely applied into various nanoparticles, such as IONPs, TiO₂ nanorods, FePt nanoparticles, Bi₂S₃ nanorods, and upconversion NaYF₄ nanoplates. Notably, the hydrophilic nanoparticles obtained through NOBF₄ treatment can undergo secondary ligand exchange with moderate anchoring groups (e.g., thiol, carboxylic acid, amino, and phosphonic acid groups). Based on this strategy, our group developed pH‐responsive RAFT polymer/iron oxide hybrid nanoparticles for pancreatic beta‐cell detection and amyloidosis mitigation.^[^ ^44^ ^]^ Overall, future advancements in the “grafting to” approach should focus on developing faster, more efficient surface modification techniques that enable scalable and environmentally friendly fabrication of hybrid nanoparticles. Additionally, integrating smart or stimuli‐responsive polymers could further enhance the adaptability and multifunctionality of hybrid nanoparticles for applications in biomedicine, catalysis, and advanced manufacturing.

#### 2.2.3 “Grafting from” Strategy

The “grafting from” approach involves the in situ polymerization of monomers directly on the surface of inorganic nanoparticles (Figure 2, Entry 7 in Table 1). In this method, chain transfer agents (CTAs) or initiators are first covalently anchored onto the nanoparticle surface, allowing polymer chains to grow directly from the nanoparticles. Unlike the “grafting to” approach, which conjugates pre‐synthesized high‐molecular‐weight polymers, the smaller molecular size of CTAs or initiators in the “grafting from” method reduces steric hindrance, leading to higher grafting density and improved polymer‐nanoparticle integration. For instance, cubic IONPs pose stabilization challenges in aqueous media owing to their high surface energy and anisotropic surface chemistry.^[^ ^45^ ^]^ To overcome it, the “grafting from” strategy was employed to conjugate CTA onto the cubic IONP surface, enabling in situ polymerization of N‐isopropylacrylamide (NIPAM) and poly(ethylene glycol) methyl ether acrylate (PEGA) monomers. This method resulted in cubic IONPs with significantly enhanced colloidal stability, ensuring better dispersion and long‐term stability in aqueous environments.^[^ ^46^ ^]^ Interestingly, the “grafting to” approach achieved better surface coverage than the “grafting from” approach at the same grafting density. This difference was attributed to the anisotropic polymerization observed in the “grafting from” approach, which led to poor polymer molecular weight distribution, ultimately affecting the uniformity of the polymer coating on the hybrid nanoparticles.^[^ ^47^ ^]^

Surface‐initiated controlled radical polymerization is a powerful method for growing polymer chains on the surface of nanoparticles. The process involves anchoring initiator molecules onto the surface, followed by polymer chain propagation under controlled radical conditions like RAFT and atomic transfer radical polymerization (ATRP). For instance, 2‐(2‐Bromoisobutyryloxy)ethyl phosphonic acid serve as an ATRP initiator grafted on IONPs.^[^ ^48^ ^]^ In this process, the phosphonic acid group acts as a high‐affinity anchor, ensuring the stable immobilization of ATRP initiators onto the hybrid nanoparticles. Afterward, ATRP polymerization of (dimethylamino)ethyl methacrylate was successfully carried out to achieve well‐defined polymers with controlled molecular weight and narrow dispersity. Moreover, Matyjaszewski's group covalently functionalized the oxide layer on the surface of eutectic gallium‐indium (EGaIn) nanodroplets with copper bromide as initiator to facilitate polymerization. EGaIn/polymer hybrid nanoparticles demonstrated high stability, excellent dispersibility, and tunable mechanical and optical properties, along with a significant reduction in the EGaIn solidification temperature—from 15 °C to as low as −80 °C.^[^ ^49^ ^]^ However, copper contamination poses a significant limitation in biomedicine, electronics, and optoelectronics, where even trace amounts of metal residues can interfere with functionality and performance.^[^ ^50^ ^]^ RAFT polymerization and copper‐free ATRP diminish these issues by utilizing a metal‐free radical initiation system.

### 2.3 Traditional Synthetic Approaches of Nanocomposites

Various fabrication methods for nanocomposites have been developed, including direct mixing, solution blending, melting, in situ polymerization, layer‐by‐layer assembly, and self‐assembly —each with its distinct advantages and limitations, Entry 8–13 in Table 1. These approaches are introduced based on their historical emergence and technical evolution. These techniques are presented in the order of their historical development and technical evolution, enabling a clear understanding of how nanocomposite processing has advanced.

The direct mixing technique, one of the earliest approaches introduced in the early 20th century, involves the mechanical blending of nanoparticles into a polymer matrix in powder or liquid form.^[^ ^51^ ^]^ Although this technique is a simple and cost‐effective approach for preliminary prototyping, fabricated composites often suffer from poor nanoparticle dispersion, and weak interactions between the nanoparticles and the polymer matrix.^[^ ^52^ ^]^ Consequently, these shortcomings result in suboptimal mechanical properties and reduced durability in load‐bearing applications. To overcome these limitations, alternative fabrication methods emerged between the 1930s and 1980s, including in situ polymerization,^[^ ^53^ ^]^ sol–gel method,^[^ ^54^ ^]^ solution blending,^[^ ^55^ ^]^ melt blending,^[^ ^55^, ^56^ ^]^ and electrospinning,^[^ ^52^ ^]^ were developed. These methods strategically incorporate nanoparticles before, during, and after polymer synthesis, thereby enhancing dispersion and interfacial interactions within the composite materials.

With the rapid advancement of nanotechnology, more sophisticated manufacturing methods have been introduced for fabricating nanocomposites. Among these, layer‐by‐layer assembly and self‐assembly techniques have attracted significant academic interest due to their ability to control material architecture at the molecular level. The layer‐by‐layer assembly technology involves the sequential deposition of alternating layers of polymers and hybrid nanoparticles onto a substrate via electrostatic interactions or chemical bonding.^[^ ^57^ ^]^ Although this is a time‐consuming and labor‐intensive process, the inherent precision of layer‐by‐layer assembly enables the formation of well‐defined hierarchical structures with uniformly distributed nanomaterials, while traditional fabrication techniques typically fail to achieve. Additionally, self‐assembly technology is a process that utilizes spontaneous interactions between molecules (e.g., hydrogen bonding, van der Waals forces, and electrostatic attractions) to construct nanocomposites ranging from the nanoscale to the macroscale.^[^ ^58^ ^]^ The self‐assembly approach is able to rapidly generate nanocomposites without external force. However, these advanced techniques suffer challenges in meeting high requirements with complicated structures, high resolution and customization.

### 2.4 Additive Manufacturing of Nanocomposites

3D printing (additive manufacturing) enables the precise, layer‐by‐layer construction of complex structures directly from computer‐aided design (CAD) models.^[^ ^64^ ^]^ Its ability to integrate nanoparticles or hybrid nanoparticles into printable matrices makes it a powerful tool for fabricating high‐resolution nanocomposites with tailored architectures. Compared to traditional manufacturing methods, 3D printing offers key advantages—on‐demand customization, reduced material waste, and spatial control over nanoparticle distribution.^[^ ^65^ ^]^ These features are especially valuable for biomedical devices, soft robotics, and responsive electronic materials, where function is closely tied to structure. Techniques such as fused deposition modelling (FDM),^[^ ^66^ ^]^ stereolithography (SLA),^[^ ^67^ ^]^ and two‐photon polymerization (TPP)^[^ ^68^ ^]^ enable scalable production of composites with diverse properties, depending on material choice and deposition mechanisms. A comparative summary of key performance indicators, including resolution, precision, and manufacturing efficiency, is presented in **Table** **2** to facilitate evaluation of different additive manufacturing techniques.

**Table 2:** Comparison of the main 3D printing techniques.

Printing category	Technique	Typical resolution [µm]	Precision [µm]	Printing rate	Surface finish	Post‐processing
Extrusion‐based printing	FDM	100–300	100–200	High	Moderate	Support removal and surface smoothing
	DIW	50–100	50–100	Medium–high	Moderate	Potential curing or drying
Vat photopolymerization	SLA	50–100	20–50	Medium	Smooth	Solvent washing and UV post‐cure
	DLP	25–50	10–30	Medium	Very Smooth	Solvent washing and UV post‐cure
	TPP	0.1–1	0.1–1	Low	Extremely Smooth	Removal of unused Resin
Powder bed fusion	SLS	≈100	20–50	Low–medium	Rough	De‐powdering, polishing, dyeing
	SLM	20–80	10–30	Low	Moderate	Heat treatment, support removal

**Note**: All listed parameters correspond to the typical performance ranges of standard commercial systems for each technique. Performance may vary depending on specific machine models, material systems, and processing conditions. FDM: Fused deposition modeling; DIW: Direct ink writing; SLA: Stereolithography; DLP: Digital light processing; TPP: Two‐photon polymerization; SLS: Selective laser sintering; SLM: Selective laser melting.

Compared to conventional synthesis approaches (e.g., bulk casting, molding, solvent‐based dispersion), 3D printing offers superior control over material architecture and functionality.^[^ ^69^ ^]^ In terms of size and shape control, 3D printing enables the fabrication of micron‐ to nanoscale features with high reproducibility and resolution, whereas traditional methods often result in batch‐to‐batch variability and limited geometric complexity. Morphology modulation is achieved via spatially controlled layer‐by‐layer deposition, allowing designers to embed gradients or compartmentalized functionalities that are difficult or impossible to achieve via blending or casting.^[^ ^70^ ^]^ On a compositional level, 3D printing supports multimaterial integration in a single fabrication step, enabling the co‐printing of soft and rigid domains or responsive elements—something traditional techniques typically require labor‐intensive, multistep assembly to accomplish.^[^ ^71^ ^]^

While initial equipment costs for 3D printing may be higher, it offers greater material efficiency, minimal waste, and faster prototyping cycles, which can substantially reduce overall manufacturing costs, particularly for customized or small‐batch production. Regarding scalability, recent advancements such as multinozzle printing, continuous flow systems, and modular printer arrays are rapidly closing the gap with traditional mass production techniques, offering a pathway toward scalable, automated manufacturing of nanocomposites with complex functionalities.^[^ ^72^ ^]^

Previously, our group summarized the integration of hybrid nanoparticles into 3D printing for biomedical applications.^[^ ^73^ ^]^ In this review, we highlight how 3D printing, when integrated with hybrid nanoparticles—either through direct mixing or surface coating strategies—enables the fabrication of functionally enhanced composites. These advanced materials offer unique capabilities that are pivotal for next‐generation biomedical devices and engineering applications.^[^ ^74^ ^]^

#### 2.4.1 Mix‐based Approaches

The incorporation of hybrid nanoparticles into resins provides a simple yet effective strategy for fabricating nanocomposites. The prepared hybrid nanoparticles are first dispersed into the resin to ensure uniform distribution, followed by 3D printing to form the final nanocomposite structures. This approach is cost‐effective and adaptable, making it suitable for rapid prototyping and large‐scale production. Depending on the formulation, hybrid nanoparticles can be integrated into liquid or solid resins, allowing for greater flexibility in processing and application.

A common strategy involves directly mixing hybrid nanoparticles with liquid monomer resins for vat polymerization (SLA, digital light processing (DLP)) and extrusion‐based printing (direct ink writing (DIW), FDM with UV‐curable resins). Several factors influence the success of 3D‐printed nanocomposites. The poor dispersibility and stability of nanoparticles can lead to agglomeration and precipitation during processing. This results in inhomogeneous nanoparticle distribution, clogging of printer nozzles, and a significant reduction in mechanical and multifunctionality performance. Instead, the use of hybrid nanoparticles modifies resin viscosity, which directly impacts flow behavior, layer spreading, and extrusion dynamics. In vat photopolymerization, excessive viscosity reduces light penetration and photopolymerization efficiency of resins, leading to incomplete curing and poor interlayer adhesion. Similarly, in extrusion‐based printing, high viscosity disrupts material flow through the nozzle, causing inconsistent extrusion, clogging, and excessive shear stress.

Additionally, hybrid nanoparticles exhibiting coloration could significantly block UV light penetration during 3D printing, thereby prolonging the curing time. Therefore, optimizing hybrid nanoparticles concentrations and properties of resins is essential to achieve 3D‐printed nanocomposites with uniform dispersion, enhanced printability, and excellent performance. Weng et al. incorporated organically modified SiO_2_ hybrid nanoparticles into SLA resins to investigate the structure‐property relationship of the resulting nanocomposites.^[^ ^75^ ^]^ 3‐(trimethoxysilyl)propyl methacrylate and (1‐hexadecyl)dimethyl allyl ammonium chloride were used as stabilizers to functionalize the surface of nanoparticles, ensuring enhanced dispersibility within aqueous SLA resins. Mechanical properties were enhanced with hybrid nanoparticle loading, reaching up to 10% w/w, while maintaining processability. In another study, the nanoparticle‐polymer interaction was investigated through a one‐pot approach, where the viscosity of the final resin can be precisely controlled by regulating the nanocomposite formation process, optimizing its printability and mechanical properties. However, this method has a limited selection of nanoparticles, which can be effectively synthesized by a one‐pot approach. Due to their intrinsic liquid properties, gallium‐based LMNPs can be successfully prepared into resins.^[^ ^76^ ^]^ In this process, bulk materials within the polymer‐based resin undergo probe sonication, facilitating the formation of spherical nanoparticles with an average diameter of ≈204 nm. The SEM analysis revealed excellent homogeneous distribution within the 3D‐printed nanocomposites and the printed nanocomposites demonstrated high‐resolution features.

In addition to liquid resins, hybrid nanoparticles can also be integrated with solid resins to produce objects using powder bed fusion‐based printing techniques. Roy et al. employed a microscale selective laser sintering (µ‐SLS) process to fabricate nanocomposites with sub‐5 µm resolution and a throughput exceeding 60 mm^3^ h^−1^.^[^ ^77^ ^]^ Polyvinylpyrrolidone was used as a stabilizing agent to synthesize Cu‐based hybrid nanoparticles, effectively preventing agglomeration. Subsequently, stabilized hybrid nanoparticles were then mixed with metal powders and subjected to sintering, resulting in the formation of metal electronic components with high resolution and conductivity. Although promising, several challenges need to be addressed. One of the key issues is that certain hybrid nanoparticles absorb or scatter laser energy, leading to inconsistent heat distribution and incomplete sintering. Additionally, organic components of hybrid nanoparticles are often highly sensitive to high temperature, making them susceptible to thermal degradation during the sintering process.

#### 2.4.2 Surface Coating

In addition to direct mixing, hybrid nanoparticles can also be conjugated on the surface of 3D‐printed objects by chemical and physical coating techniques. Unlike mix‐based approaches, surface coating is a post‐printing method that applies hybrid nanoparticles after the object has been successfully printed. The surface coating process places greater emphasis on coating thickness and homogeneity, rather than on the dispersion and agglomeration of hybrid nanoparticles within the solution. It also provides strong interfacial adhesion by introducing functional groups that chemically or physically bond with the surrounding polymer matrix, reducing phase separation and enhancing mechanical properties. In chemical coating, organic components of hybrid nanoparticles are conjugated to 3D‐printed constructions via chemical reactions, such as click chemistry, polymerization, and sol–gel coating. Chiappone et al. reported an innovative approach to enhance the properties of 3D‐printed nanocomposites by covalently coating silica nanoparticles (a building block of hybrid nanoparticles) onto their surfaces using a post‐printing sol–gel process.^[^ ^78^ ^]^ The sol–gel process, a wet chemical technique that converts a solution into a gel before drying it into a solid material, enables the in situ generation of silica nanoparticles, significantly enhancing the surface hardness of coatings.^[^ ^79^ ^]^ Notably, Young's modulus of the final product is twice that of 3D‐printed objects without coating, demonstrating a significant increase in mechanical performance.

Physical coating generates a protective or functional layer onto the surface of 3D‐printed objects through physical deposition methods, such as physical vapor deposition, dip coating, and cold spraying.^[^ ^80^ ^]^ These techniques precisely control film thickness and composition to enhance surface properties—including mechanical durability, electrical conductivity, biocompatibility, and environmental resistance. Zhu et al. physically coated MXene, a class of two‐dimensional metal carbides, onto FDM‐printed carbon black‐based electrodes for enhanced electrical conductivity from 20.8 to 30.2 mF cm^−2^ at a current density of 0.1 mA cm^−2^.^[^ ^81^ ^]^ Meanwhile, the electrodes demonstrated a stable cycle life, retaining 95% of their capacitance after 10 000 cycles. Physical methods exhibit less stability between hybrid nanoparticles and printed objects than chemical coating due to weaker adhesion and lower resistance to environmental stressors.

In addition to conventional hybrid nanoparticles, organic‐origin nanomaterials such as covalent organic frameworks (COFs) and carbon dots (CDs) have increasingly been recognized for their ability to function as inorganic analogues in the fabrication of hybrid nanoparticles, owing to their inorganic‐like structural features and functional properties. Despite being composed of organic elements, COFs exhibit crystalline architectures, high thermal and chemical stability, and tunable porosity, characteristics typically attributed to inorganic materials such as zeolites or mesoporous silica.^[^ ^82^ ^]^ Likewise, CDs possess size‐dependent photoluminescence, excellent chemical robustness, and favorable electronic conductivity, enabling them to emulate the behavior of inorganic quantum dots.^[^ ^83^ ^]^ The advancement of 3D printing has opened new avenues for the incorporation of these organic nanoparticles into complex architectures.

### 2.5 Properties and Performance of 3D‐Printed Nanocomposites

#### 2.5.1 Key Microstructural Factors Influencing Nanocomposites

The microstructural characteristics of nanoparticles—most notably the dispersion quality, degree of nanoparticles aggregation, orientation of the fillers, and the filler–matrix interface—play a decisive role in determining their mechanical, electrical, thermal, and overall functional performance of nanocomposites.^[^ ^84^ ^]^ Well‐dispersed nanoparticles can strengthen nanocomposites more effectively and improve properties such as heat resistance, stiffness, and durability. Incorporating graphene into polyethylene matrices has been demonstrated to significantly enhance the thermal conductivity of nanocomposites.^[^ ^85^ ^]^ It is primarily attributed to the formation of efficient heat conduction pathways facilitated by well‐dispersed graphene layers within the polymer matrix. Conversely, poor dispersion or aggregation, as observed in polypropylene/silica systems, leads to stress concentrations and premature mechanical failure. Specifically, the use of unmodified silica particles or the absence of compatibilizers results in clustering within the polypropylene matrix, correlating with weak mechanical integrity.^[^ ^86^ ^]^ Moreover, the orientation of anisotropic fillers, such as CNTs, can be strategically controlled to tailor directional properties. When aligned, these fillers significantly enhance mechanical strength, electrical conductivity, and thermal transport along the alignment direction.^[^ ^87^ ^]^ For example, in electrospun fiber‐based composites, aligning CNTs along the fiber axis significantly reduces the percolation threshold to ≈0.0031 vol%, which is over an order of magnitude lower than the 0.034 vol% observed in systems with randomly oriented CNTs. Aligned CNTs of 0.3 wt% have also been shown to enhance the elastic modulus and fracture toughness by ≈40% and 50%, respectively, demonstrating the substantial mechanical advantages achieved through controlled filler orientation. Characterization techniques like transmission electron microscopy and atomic force microscopy are essential tools for analyzing microstructural features. Processing techniques, including shear mixing, extrusion‐induced alignment, and ultrasonication, are commonly utilized to promote uniform dispersion, consistent distribution, and desired filler orientation, all of which are vital for optimizing nanocomposite performance.

However, even with optimal dispersion and loading, inadequate interfacial bonding between the nanoparticles and the polymer matrix can lead to inefficient stress transfer, localized deformation, and ultimately poor mechanical and functional properties. Surface chemistry serves as a powerful tool to modulate interfacial interactions, directly impacting the structural integrity and overall performance of nanocomposites.^[^ ^88^ ^]^ For example, amine‐functionalized silica nanoparticles have displayed strong interfacial adhesion with polyurethane matrices, leading to marked improvements in both mechanical strength and thermal resistance.^[^ ^89^ ^]^ Similarly, acid‐ or plasma‐treated CNTs have shown improved dispersion and stress transfer in thermoplastic systems due to the introduction of oxygen‐containing functional groups, which foster stronger interfacial bonding.^[^ ^90^ ^]^ Beyond reinforcement, surface engineering also enables application‐specific properties. For instance, hydroxyapatite nanoparticles functionalized with carboxyl or hydroxyl groups have been successfully embedded into biodegradable polymers to create bioactive scaffolds with improved cell adhesion and osteoconductive properties, making them ideal candidates for bone tissue engineering.^[^ ^91^ ^]^ Thus, the rational design of surface chemistry is essential not only for enhancing material performance but also for expanding the functional versatility of nanocomposites across a broad range of advanced applications.

In additive manufacturing, the microstructural characteristics of nanoparticles, particularly the dispersion, aggregation, orientation and interface between nanoparticles and resins, play a fundamental role in determining mechanical, thermal, electrical, and functional properties of 3D‐printed nanocomposites.^[^ ^92^ ^]^ Among these factors, achieving uniform dispersion while minimizing aggregation is a fundamental challenge in the fabrication of high‐performance 3D‐printed nanocomposites. Homogeneous dispersion ensures nanofillers are evenly distributed throughout the polymer matrix. However, due to their high surface energy and strong van der Waals interactions, nanoparticles, such as CNTs, graphene, or metal oxides, tend to aggregate during mixing and printing. In vat photopolymerization systems, poorly dispersed nanofillers can scatter or absorb UV light, impairing curing depth and reducing print resolution.^[^ ^93^ ^]^ In extrusion‐based techniques, nanofiller agglomeration may lead to nozzle clogging and nonuniform material flow, resulting in structural defects and compromised print quality. To address these issues, various strategies have been employed to promote dispersion and prevent aggregation, including surface functionalization, ultrasonication, the addition of dispersing agents, and in situ polymerization during the printing process.

Extrusion‐based printing techniques, like FDM and DIW, naturally introduce shear forces during the printing process, which induce the alignment of high‐aspect‐ratio hybrid nanoparticles along the direction of material deposition. This orientation is highly beneficial for enhancing anisotropic properties such as tensile strength, electrical conductivity, and thermal transport of 3D‐printed nanocomposites. FDM‐printed nanocomposites incorporating graphene nanoplatelets (GNPs) in linear low‐density polyethylene have demonstrated enhanced in‐plane electrical conductivity and mechanical strength, primarily due to the shear‐induced alignment of GNPs along the extrusion direction during printing.^[^ ^94^ ^]^ Notably, the resulting 3D‐printed nanocomposites exhibited a significant improvement in through‐plane thermal conductivity, reaching 3.43 W·m^−1^·K^−1^ along the printing direction. This value markedly surpasses that of both neat polymer materials (0.40 W·m^−1^·K^−1^) and conventionally melt‐compounded nanocomposites (1.98 W·m^−1^·K^−1^). Additionally, the interface between nanoparticles and the polymer matrix influences the mechanical robustness, stress tolerance, and long‐term reliability of 3D‐printed nanocomposites.^[^ ^95^ ^]^ A strong interface facilitates efficient stress transfer and load distribution, enabling the nanocomposite to resist crack initiation and propagation under mechanical or environmental stress. Weak interfacial adhesion, nanofillers may detach or form weak points that lead to structural failure. A recent study has demonstrated that boron nitride nanosheets (BNNS) incorporated into epoxy‐based resins via stereolithography significantly enhanced thermal conductivity and mechanical strength, owing to improved interfacial bonding achieved through amine surface functionalization.^[^ ^96^ ^]^ This improvement is attributed to the fact that hexagonal boron nitride (hBN) layers dispersed along boron nitride nanotubes (BNNTs) create an irregular surface topology, which helps reduce agglomeration by maintaining sufficient spacing and minimizing van der Waals attractions. Additionally, the hBN decoration enhances interfacial interaction by enabling mechanical interlocking with the matrix while preserving the polarity, further improving compatibility and load transfer efficiency. In conclusion, the structural and functional properties of 3D‐printed nanocomposites are profoundly influenced by microstructural parameters, including the uniformity of dispersion, the extent of aggregation, the spatial orientation, and the interfacial interactions. A comprehensive understanding and precise modulation of these factors are imperative for optimizing composite performance and are expected to play a pivotal role in the advancement of high‐performance, application‐specific nanocomposites in the context of additive manufacturing.

#### 2.5.2 Mechanical Properties

**Figure 3:**
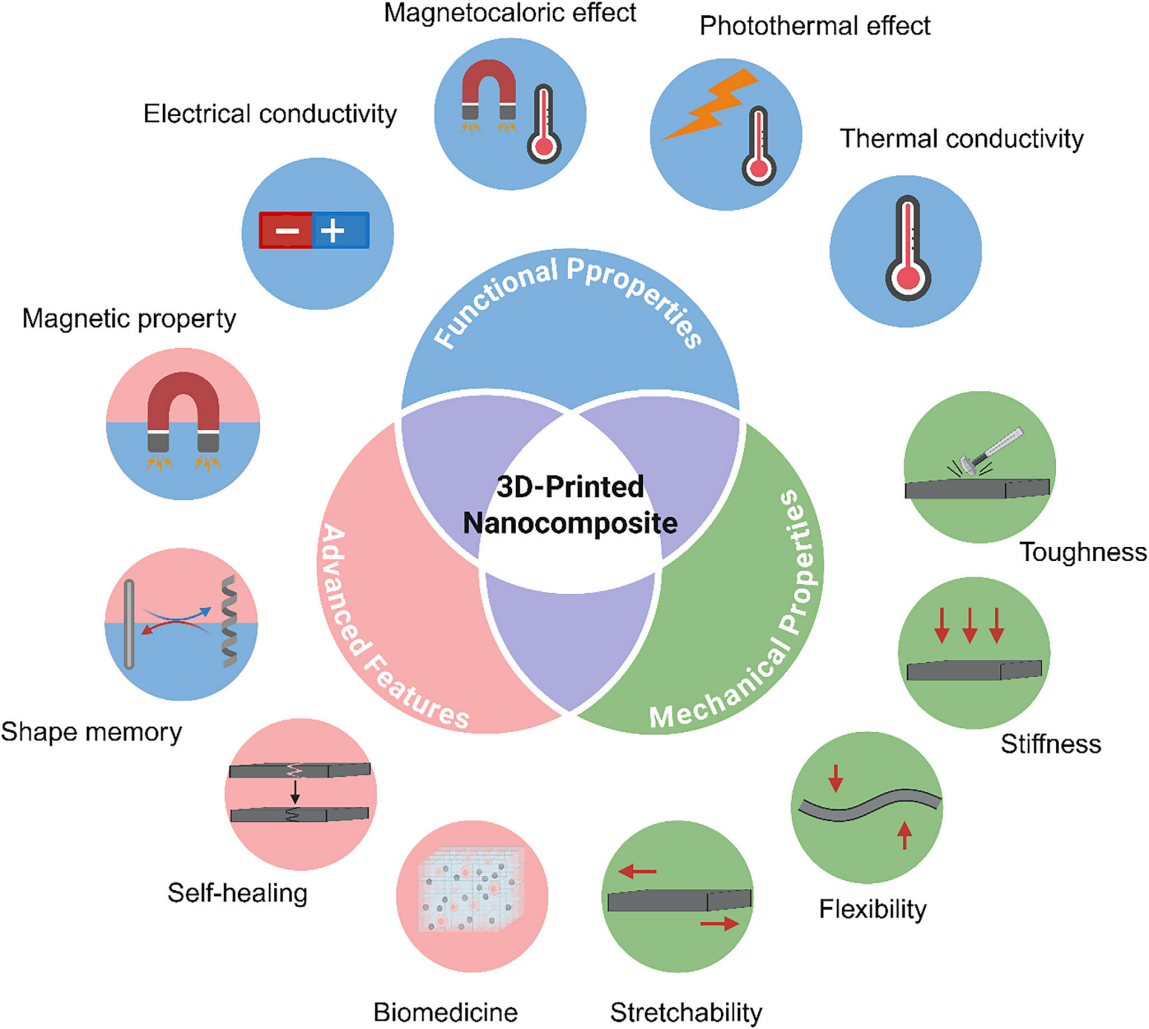
The main properties of 3D‐printed nanocomposites are achieved by integrating hybrid nanoparticles into the matrix to enhance mechanical strength, functional performance, and other material characteristics. Created with BioRender.com.

Integrating hybrid nanoparticles into 3D‐printed objects enhances their mechanical properties, improving their strength, stiffness, and dimensional stability (**Figure** **3**). By increasing the interfacial area, hybrid nanoparticles facilitate efficient stress transfer and uniform stress distribution, reducing micro‐cracking and enhancing both tensile and flexural strength.^[^ ^97^ ^]^ Such advantages are particularly valuable in dentistry and bone defect treatment, where high‐stress tolerance and long‐term durability are essential.

One key advantage of incorporating hybrid nanoparticles into 3D‐printed nanocomposites is their ability to enhance toughness and stiffness by introducing inter‐filler interactions, which create nanoscale barriers that deflect and branch propagating cracks, thereby dissipating energy and reducing the risk of fracture.^[^ ^98^ ^]^ Meanwhile, the high surface area enhances interfacial adhesion with the matrix, distributing stress more effectively and resisting crack formation. GO is a popular nanosheet to improve the toughness, strength and ductility of composites. Its strong interfacial bonding, high aspect ratio, and crack‐bridging ability enable efficient stress transfer and resistance to fracture.^[^ ^99^ ^]^ Manapat et al. reported that incorporating just 1.0 wt% GO into SLA‐printed nanocomposites resulted in a remarkable 673.6% increase in tensile strength, alongside a substantial improvement in Young's modulus from 717.4 ± 41.94 to 1229 ± 91.75 MPa.^[^ ^99^ ^]^ Markandan and Lai also demonstrated the incorporation of a low concentration of GO (0.1 wt%) increased tensile strength to 45 MPa and improved Young's modulus by 30%.^[^ ^100^ ^]^ Low concentrations of GO significantly enhance load‐bearing capacity and impact resistance, showcasing the remarkable efficiency of GO in achieving substantial mechanical improvements with minimal material addition.

Moreover, 3D‐printed nanocomposites containing titanium further improve mechanical properties, making them highly suitable for high‐performance applications.^[^ ^101^ ^]^ In contrast to carbon‐based nanomaterials, their high density provides superior load‐bearing capacity and structural reinforcement. Lin et al. demonstrated the effectiveness of dispersed titanium carbide (TiC) nanoparticles in reinforcing aluminum through laser additive manufacturing.^[^ ^102^ ^]^ With a high TiC nanoparticle density (up to 35 vol%), the mechanical testing of the composite exhibited an exceptional yield strength of up to 1000 MPa, plasticity exceeding 10%, and a Young's modulus of ≈200 GPa, highlighting its potential for high‐performance structural applications. Enhanced performance arises from the uniform dispersion of high‐density nanoparticles, strong interfacial adhesion between the TiC nanoparticles and the aluminum matrix, and the formation of ultrafine grain structures, which collectively contribute to improved mechanical properties. Unlike rigid nanoparticles, hybrid nanoparticles with soft and liquid features can be used to reduce the mechanical strength and stiffness of 3D‐printed nanocomposites. Zhang et al. reported that incorporating 2 wt% of LMNPs into an SLA‐printed polymer matrix led to a significant reduction in tensile strength from 18.00 ± 1.76 to 6.37 ± 0.72 MPa and elastic modulus from 434.38 ± 20.63 to 173.88 ± 13.95 MPa, highlighting the softening effect of liquid metal nanoparticles on the composite structure.^[^ ^76^ ^]^ This behavior is attributed to the soft and pliable nature of room‐temperature LMNPs, which offer less resistance to polymer chain motion than rigid nanoparticles, thereby reducing stress transfer efficiency and leading to a decline in mechanical strength and stiffness.

In addition to toughness and stiffness, the incorporation of hybrid nanoparticles also changes the flexibility and stretchability of 3D‐printed nanocomposites.^[^ ^103^ ^]^ Specifically, GO increases surface roughness, stiffness and flexibility, making 3D‐printed nanocomposites an excellent material for wearable devices and electronic applications. This improvement is attributed to the high inherent elastic modulus and strength of GO nanoplatelets, strong interactions with the polymer matrix, and excellent uniform dispersion within the resin.^[^ ^104^ ^]^ For example, 0.5 wt% GO was incorporated into thermoplastic polyurethane and poly(lactic acid) resins to successfully fabricate GO‐based nanocomposites using an FDM printer.^[^ ^105^ ^]^ The addition of GO significantly enhanced the mechanical properties and thermal stability of the nanocomposites, demonstrating its potential as a biomaterial scaffold for tissue engineering applications. Beyond GO, cellulose nanocrystals (CNCs) can improve the mechanical resilience and flexibility of nanocomposites due to their high aspect ratio and hydrogen bonding interactions with the polymer.^[^ ^106^ ^]^ Justo et al. demonstrated the effective incorporation of CNCs into nylon materials using an FDM 3D printer,^[^ ^106a^ ^]^ Where the addition of 10 wt% CNCs resulted in a 34% increase in elastic strength in the longitudinal direction and a 13% improvement in the transverse direction compared to the pure polymer matrix.

Another significant benefit of excellent flexibility is its potential for enhancing recyclability, as highly flexible materials can better withstand repeated mechanical processing without degradation. The addition of aligned nanofibers within 3D‐printed nanocomposites significantly enhances their durability, enabling them to withstand over 800 000 mechanical load cycles without deterioration.^[^ ^107^ ^]^ Overall, incorporating hybrid nanoparticles into 3D‐printed nanocomposites effectively controls toughness, stiffness, flexibility, and durability, making them suitable for high‐performance applications.

#### 2.5.3 Functional Properties

Some functional hybrid nanoparticles possess thermal, electrical, and magnetic properties. Integrating them into 3D printing systems enhances nanocomposites with additional functionalities, enabling innovative applications in electronics, biomedicine, and structural components (Figure 3).

Developing 3D‐printed composites with high thermal conductivity is essential for effective thermal dissipation, which directly impacts the performance, lifespan, and reliability of devices.^[^ ^76^ ^]^ For instance, DLP‐printed acrylate composites incorporating 30 wt% aluminum nitride (AlN) demonstrated significantly enhanced heat conduction.^[^ ^108^ ^]^ Compared to pure acrylate resins, AlN‐based nanocomposites enhance in‐plane thermal conductivity by 477% (1.43 W m^−1^·K^−1^) and through‐plane conductivity by 350% (0.35 W m^−1^·K^−1^). This improvement is attributed to the uniform dispersion of AlN particles, which create efficient thermal transport pathways within the layered polymer matrix.

Additionally, by incorporating photothermal nanoparticles enables the nanocomposites absorb light energy leading to a controlled temperature increase. Among these, gold‐based nanorods are widely used as photothermal agents owning to their localized surface plasmon resonance.^[^ ^109^ ^]^ This property makes them valuable in applications such as biomedical therapy, smart materials, and energy harvesting. Skillin et al. used FDM to print liquid crystalline elastomer nanocomposites containing gold nanorods.^[^ ^110^ ^]^ Photothermal experiments displayed that adding only 0.1 wt% gold nanorods rapidly increased the material's temperature to 60 °C under neaar‐infrared (NIR) light irradiation (808 nm, 1.7 W cm^−2^) for 30 s, achieving rapid heating rates over 150 °C s^−1^.

The magnetocaloric effect (MCE) is an advanced thermal management strategy that enables rapid, reversible, and tunable temperature control through an external magnetic field. Compared to thermal and photothermal effects, MCE offers contactless and light‐independent heat modulation, making it more precise and adaptable. Furthermore, the remote generation of localized hyperthermia using a magnetic field and IONPs allows for controlled thermal conductivity in targeted areas, making it highly effective for biomedical applications. Recent advancements have demonstrated the direct 3D printing of IONP‐embedded polylactic acid (PLA) and hydroxyapatite (HA) scaffolds.^[^ ^111^ ^]^ Magnetocaloric experiments exhibited that nanocomposites enable precise temperature control, from moderate hyperthermia (42–50 °C) to ablation (50–55 °C) under a 330 kHz alternating magnetic field, making them ideal for magnetic hyperthermia therapy.

In addition to thermal properties, the incorporation of metallic hybrid nanoparticles significantly increases the electrical conductivity of 3D‐printed nanocomposites by forming efficient charge transport networks and minimizing electron scattering.^[^ ^112^ ^]^ Efficient electrical conductivity enables rapid signal transmission, precise control, and reduced energy loss, making it particularly advantageous for applications in cell signal processing, wearable electronics, energy storage, and high‐performance sensors. Boularaoui et al. prepared high‐conductivity bioinks by adding 0.1 mg mL^−1^ MXene nanosheets or gold nanoparticles,^[^ ^113^ ^]^ achieving conductivity values of 0.8 ± 0.07 and 0.9 ± 0.12 S m^−1^, respectively, compared to 0.3 ± 0.06 S m^−1^ for pure gelatin methacryloyl (GelMA). This improvement facilitated better cell‐to‐cell communication and promoted myoblast differentiation, leading to >97% viability in printed skeletal muscle tissues.

Beyond biomedical applications, highly conductive 3D‐printed nanocomposites are promising for electronics and sensor technologies. Shao et al. fabricated flexible wireless electronics using additive‐free titanium carbide (Ti₃C₂Tx) MXene‐based inks via programmable extrusion printing. The inks have achieved metallic conductivity (≈6900 S cm^−1^) in the ultrafine‐printed tracks (3 µm line gap) without annealing.^[^ ^114^ ^]^ Similarly, Kwon et al. developed piezoresistive sensors by integrating GO and silver nanoparticles (AgNPs) into a polyurethane matrix.^[^ ^115^ ^]^ The sensor exhibited stain detection exceeding 160% with a high gauge factor of 48.2, far surpassing conventional stretchable sensors. The synergy between GO and AgNPs enhanced electrical conductivity and improved strain‐sensing performance through resistance modulation. Notably, graphene reinforcement significantly prolonged sensor lifespan, demonstrating excellent stability over 500 cycles, underscoring its potential for wearable applications in human motion detection and interactive devices.

3D‐printed objects containing magnetic‐responsive nanoparticles can be remotely actuated by external magnetic fields, enabling precise, contactless control. Magnetic fields can penetrate nonmagnetic materials, including biological tissues, water, and polymers, allowing for rapid and controlled motion. Specifically, neodymium magnets are the strongest commercially available permanent magnets, composed of neodymium, iron, and boron.^[^ ^116^ ^]^ Li et al. fabricated magnetic‐responsive soft robots using neodymium particle‐based resin, achieving a magnetization resolution of 350 µm.^[^ ^117^ ^]^ High resolution enables precise control under external magnetic fields, as allowing locomotion such as rolling, gripping, swimming, and walking, even in constrained environments. An innovative indirect fabrication strategy was introduced, leveraging controlled desiccation to enable the printing of 3D hydrogel scaffolds at the macroscopic scale, which subsequently shrink into microscale architectures upon drying.^[^ ^118^ ^]^ Notably, the hydrogel structures functioned as magnetically responsive microrobots capable of precise actuation under external magnetic fields, including rotational motion and directed locomotion along predefined paths. Beyond actuation, 3D printing offers customized therapeutic solutions, making it a powerful tool for personalized medicine. Ceylan et al. demonstrated this potential by developing multiresponsive micro swimmers and micro rollers using TPP.^[^ ^119^ ^]^ By incorporating magnetic nanocomposites derived from patient biomaterials, such as blood plasma, serum albumin, and platelet lysate, they created patient‐specific micromachines that effectively respond to time‐variant magnetic fields, enabling torque‐driven motion. Additionally, these micromachines exhibit pH‐responsive two‐way shape memory behavior, allowing for controlled cargo delivery and release. Magnetic responsiveness enables multifunctional, high‐precision control and enhanced responsiveness, expanding the potential of 3D‐printed nanocomposites in soft robotics, biomedical engineering, and smart materials.

Increasingly, hybrid nanoparticles exhibit multiple functionalities, enabling 3D‐printed nanocomposites to respond to different stimuli for enhanced performance. Compared to single‐responsive materials, multiple‐stimuli‐responsive nanocomposites dynamically adjust to environmental changes such as temperature, pH, light, and magnetic fields, ensuring greater control and versatility. Wang et al. demonstrated next‐generation magnetic soft robots capable of sensing multiple stimuli, including temperature with a linear response of 3.383 kΩ °C^−1^, electrochemical changes with a detection limit as low as 0.036 mm, and tactile recognition of materials such as wood, iron, and stone.^[^ ^120^ ^]^ These magnetic soft bodies were fabricated by incorporating various hybrid nanoparticles infused inks, where neodymium‐iron‐boron enabled magnetic actuation and deformation, multiwalled carbon nanotubes facilitated tactile and electrochemical sensing, and reduced GO‐enhanced temperature sensing.

#### 2.5.4 Advanced Functions on Self‐Healing, Shape Memory, and Biomedical Functionalities

Hybrid nanoparticles play a crucial role in broadening the application scope of 3D‐printed nanocomposites by introducing advanced functionalities such as self‐healing, shape memory, and antibacterial effects. One of the major challenges in composite materials is structural failure due to microcracks that develop over time.^[^ ^121^ ^]^ Self‐healing materials address this issue by autonomously repairing damage, thereby preventing failure and extending material lifespan. Wu et al. developed a chitosan/carbon nanotube‐based nanocomposite with electrical conductivity and self‐healing properties for electronic applications.^[^ ^122^ ^]^ Exposure to water vapor enables rapid self‐healing within 10 s, restoring both electrical conductivity and mechanical integrity. After healing, the chitosan/carbon nanotube fibers recovered to 1450 S m^−1^, aided by the polymer's swelling and chain mobility. This combination of properties enables the fabrication of highly tunable microstructure fibers, making them well suited for strain gauges and humidity sensors.

Similarly, the shape memory effect has been extensively explored in 3D‐printed nanocomposites, allowing for reversible shape transformations in response to external stimuli. These materials can retain their original shape and recover when exposed to specific triggers such as heat, mechanical stress, or magnetic fields, making them ideal for smart materials and responsive systems. Wan et al. demonstrated that 3D‐printed nanocomposites incorporating 20 wt% IONPs exhibited an advanced shape memory effect, enabling quintuple shape transformations when subjected to sequential heat and magnetic field stimuli.^[^ ^123^ ^]^ The transformation was driven by the thermal transitions of the polymer matrix and the magnetic responsiveness of hybrid nanoparticles, allowing for precise, programmable deformations. Overall, self‐healing nanocomposites offer improved longevity and reliability, while shape memory materials enable highly dynamic and programmable deformations.

It also enhances the biomedical functionality of 3D‐printed nanocomposites.^[^ ^73^ ^]^ AgNPs and zinc oxide (ZnO) nanoparticles, known for their antibacterial properties, can be integrated into 3D‐printed materials to address antibiotic resistance and mitigate the antibiotic‐related side effects.^[^ ^124^ ^]^ AgNPs exhibit multiple antibacterial mechanisms leading to bacterial cell death, such as bacterial membrane disruption, reactive oxygen species (ROS) generation, silver ion (Ag⁺) release that inactivates enzymes, DNA replication interference, and biofilm inhibition. Alizadehgiashi et al. fabricated a chitosan‐based hydrogel incorporating AgNPs for wound dressing applications.^[^ ^125^ ^]^ The hydrogel demonstrated a controlled release of Ag⁺ ions, resulting in a potent antibacterial effect against *Staphylococcus aureus* (*S. aureus*) and *Pseudomonas aeruginosa* (*P. aeruginosa*), by distinct zones of inhibition with an average width of 17 mm. ZnO nanoparticles exert antibacterial effects through photocatalytic activity and electrostatic interactions with bacterial membranes, leading to membrane damage and ROS production. Das et al. investigated the antibacterial properties of a microextrusion‐printed borophene/zinc oxide (BZ/ZnO) nanocomposite hydrogel, exhibiting a minimum inhibitory concentration of 250 µg mL^−1^.^[^ ^126^ ^]^ Additionally, 3D‐printed nanocomposites demonstrate excellent performance in tissue engineering by promoting cell growth, differentiation, and tissue regeneration through enhanced biocompatibility, structural support, and bioactive signaling.^[^ ^127^ ^]^ The integration of hybrid nanoparticles into 3D‐printed nanocomposites significantly enhances functionality, durability, and adaptability. These advancements not only expand the possibilities of 3D‐printed smart materials but also pave the way for next‐generation applications in electronics, robotics, and biomedical engineering.

## 3 Applications of 3D‐Printed Nanocomposites

So far, 3D‐printed nanocomposites have enabled significant advancements in soft robotics, biomedical implants, drug delivery systems, wearable sensors, and energy storage devices, pushing the boundaries of nanocomposite performance and functionality. The ability to precisely design and fabricate nanocomposite structures with microscale resolution and complex geometries paves the way for next‐generation technologies in healthcare, aerospace, and sustainable manufacturing. This section primarily highlights emerging techniques and methodologies in 3D‐printed nanocomposites containing hybrid nanoparticles, emphasizing technological advancements rather than merely outlining their applications.

### 3.1 Biomedical Applications

**Figure 4:**
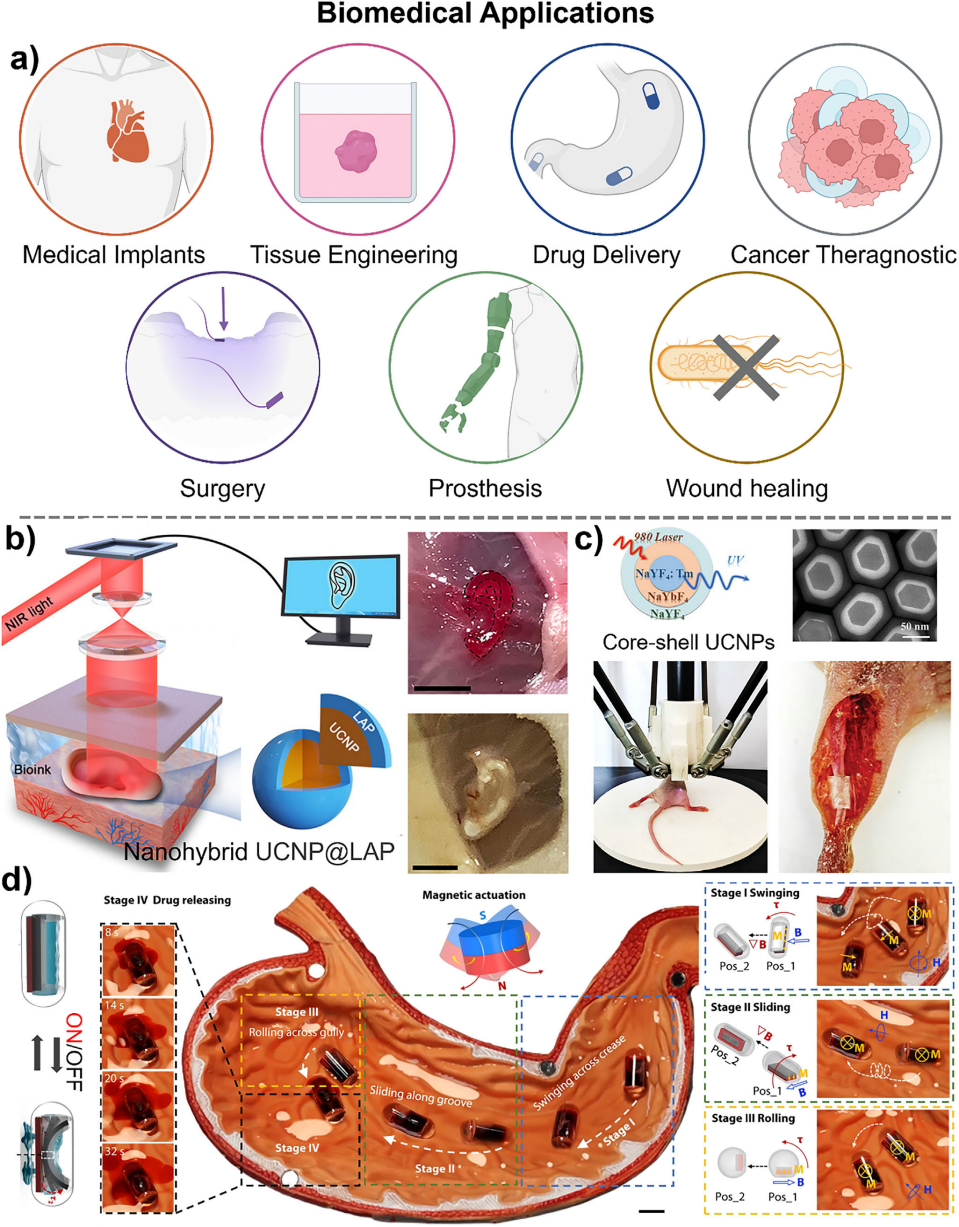
Biomedical applications and current developments of 3D‐printed nanocomposites with hybrid nanoparticles. a) Key biomedical applications of 3D‐printed nanocomposites. Created with BioRender.com. b) Schematic representation of noninvasive 3D bioprinting for in vivo living tissue fabrication using bioink containing UCNP@LAP nanoinitiators. An ear‐shaped construct was printed subcutaneously in BALB/c nude mice and observed after 1 month. Scale bar: 5 mm. Reproduced with permission.^[^ ^128^ ^]^ Copyright 2012, American Association for the Advancement of Science. c) Schematic of noninvasive bone fixation enabled by UCNP‐assisted 3D bioprinting in vivo. Reproduced with permission.^[^ ^129^ ^]^ Copyright 2024, Wiley‐VCH. d). A schematic diagram of a soft valve controlled by an external magnetic field shows targeted drug delivery with a permanent magnet and drug release in a stomach model using a 30 Hz, 30 mT high‐frequency magnetic field. Reproduced under the terms of the CC‐BY Creative Commons Attribution 4.0 International license (https://creativecommons.org/licenses/by/4.0).^[^ ^130^ ^]^ Copyright 2024, The Authors, published by Springer Nature.

3D‐printed composites have revolutionized biomedicine, driving advancements in medical implants, tissue engineering, drug delivery, and prosthetics (**Figure** **4**a).^[^ ^131^ ^]^ Integration of hybrid nanoparticles overcomes limitations of traditional 3D‐printed materials, such as weak mechanical strength, and low bioactivity. The synergy between 3D printing and nanotechnology enables the fabrication of complex, functional biomaterials, offering improved mechanical robustness, controlled porosity, and enhanced biological activity.^[^ ^132^ ^]^ In bone tissue engineering, 3D‐printed scaffolds incorporating nanoparticles have superior mechanical properties and regeneration.^[^ ^133^ ^]^ ZnO integrated 3D‐printed hydrogels significantly improve the mechanical properties of the bone constructs, accelerating tissue regeneration, and promoting bone and vascular reconstruction in defect areas.^[^ ^134^ ^]^ In addition, 3D‐printed nanocomposites have enabled advanced drug delivery systems for on‐demand drug release. Hybrid nanoparticles also enhance bioactivity, site‐specific delivery to diminish the dosage of administration.^[^ ^135^ ^]^ Despite its numerous potential, several challenges remain. Ensuring the long‐term stability and biocompatibility of nanocomposites is critical for their clinical translation.^[^ ^136^ ^]^ As such achieving precise control over material behavior, degradation rates, and real‐time adaptability in complex in vivo environments is the critical frontier in 3D bioprinting.

In parallel, modern medicine is advancing toward the development of minimally invasive approaches that reduce the large incisions of traditional open surgeries, lower postoperative morbidity, and shorten recovery times.^[^ ^137^ ^]^ Currently, there are three key strategies for cutting‐edge biomedical applications of 3D‐printed nanocomposites: 1) noninvasive in vivo printing for direct tissue engineering repair, 2) small‐scale nanocomposites for precision diagnosis and minimally invasive therapies, and 3) multifunctional nanocomposites for synergistic effects. 3D printing techniques, including inkjet printing, extrusion printing, light‐assisted printing, and laser direct writing, have been employed to manufacture customized and personalized medical implants for diagnostic and therapeutic applications.^[^ ^138^ ^]^ Traditionally, the implementation of 3D‐printed implants requires fabrication followed by surgical implantation, a complex process that can lead to tissue damage, increased infection risk, and prolonged patient recovery.

To address these challenges, the development of noninvasive 3D printing is crucial. This technology enables the direct fabrication of structures within affected areas commonly involved in surgical interventions or the in situ printing of biomaterials directly onto exposed tissue surfaces.^[^ ^139^ ^]^ Unlike traditional printing, where constructs are fabricated externally and then implanted, in vivo 3D printing enables the real‐time deposition of biomaterials at the defect site by using a minimally invasive approach.^[^ ^128^ ^]^ In this technique, the limitation of using UV or blue light as an initiating energy source in SLA and DLP is their poor tissue penetration capacity. Although NIR light exhibits more optically transparent and deeper light penetration, its relatively low energy is insufficient to activate photoinitiators for printing.^[^ ^140^ ^]^ To address this challenge, Chen and colleagues developed UCNPs with a core‐shell structure as nano initiators for noninvasive in vivo 3D bioprinting (Figure 4b), through which an ear‐structured scaffold was 3D printed for real‐time imaging.^[^ ^128^ ^]^

UNCPs exhibit the unique feature to upconvert long‐wavelength radiation (980 nm) into short‐wavelength visible light or UV emissions (345 and 361 nm). This property allows UCNPs to utilize the deep tissue penetration capability of NIR light while simultaneously generating high‐energy UV emissions to activate photoinitiators for photopolymerization.^[^ ^129^ ^]^ After exposure to NIR light for 15 s, the precursor solution containing 15 wt % GelMA and 1 wt % UCNPs‐based nanoinitiators successfully polymerized into hydrogels, demonstrating high photopolymerization efficiency. Furthermore, in vivo 3D bioprinting experiments showed that an ear‐shaped construct was successfully printed noninvasively within 20 s of NIR exposure. The printed ears‐maintained shape and structural integrity after 1 month, indicating promising in vivo tissue engineering by noninvasive bioprinting. Zhang et al. also utilized the upconversion‐based 3D bioprinting for in vivo fracture repair.^[^ ^129^ ^]^ Using CT‐guided 3D reconstruction, a bioink solution containing UCNPs was injected into the fracture site in the mice, and polymerized in situ under computer‐controlled NIR exposure, enabling precise scaffold formation without surgery (Figure 4c).

Overall, in vivo 3D printing technology has become a promising technique for minimally invasive regenerative medicine, enabling deep tissue molding without the need for surgical implantation. These innovations pave the way for advanced biomedical applications in tissue engineering, wound healing, and organ regeneration. Several limitations need to be addressed in future research. For example, biological tissues in living organisms undergo continuous motion during the printing process, which can disrupt the precise deposition of bioinks, leading to misalignment or structural deformation. Additionally, light scattering and absorption in biological tissues reduce the effective resolution of NIR and UV‐based bioprinting, posing challenges for achieving precision and consistency in dynamic in vivo environments.

Small‐scale nanocomposites are revolutionizing the development of drug delivery systems. The advanced drug delivery system enables precise targeting and controlled release, maintaining efficacy and minimizing systemic side effects within dynamic in vivo environments. Its microscale design improves bioavailability, allowing drugs to penetrate biological barriers more effectively and reach specific tissues or organs. Ceylan et al. utilized two‐photon‐based 3D printing technology to fabricate a microswimmer drug delivery system (20 µm in length and 6 µm in width), composed of GelMA and superparamagnetic IONPs.^[^ ^141^ ^]^ Microswimmers can be fabricated in various geometric shapes using highly precise light‐based 3D printing methods, ensuring reproducibility at the microscale.^[^ ^142^ ^]^ The encapsulation of the IONPs enables magnetic steering control of the microswimmers, allowing the microswimmers to navigate through biological environments with precise motion patterns, including wobbling, corkscrew, and step‐out motions. Notably, the microswimmers were degraded by the disease‐marker enzyme MMP‐2, triggering accelerated drug release at the tumor site for cancer treatment. Unfortunately, the lack of in vivo studies has limited its validation and potential for clinical translation. Sun et al. developed small‐scale capsules containing neodymium‐iron‐boron (NdFeB) nanomaterials, offering magnetically controlled responses and multifunctional fidelity in a rabbit's stomach.^[^ ^130^ ^]^ Capsules operate through competitive interactions between magnetic gradient force and magnetic torque, allowing them to remain self‐closed in the absence of a magnetic field and open when a magnetic field is applied (Figure 4d). The multimodal motion was demonstrated in a human stomach anatomical model, exhibiting swinging, sliding, and rolling capabilities in complex environments. More importantly, in vivo experiments confirmed the feasibility of these capsules for drug delivery in a rabbit's stomach using a permanent magnet. After injection, capsules rolled directionally under a rotating magnetic field within 0–4 s. At 34.5 s, a high‐frequency magnetic field enabled drug exchange with gastric juice, leading to drug release within 22 s over an area of ≈302.9 mm^2^. These animal studies demonstrated that magnetically driven capsules are a feasible and effective system for multifunctional and targeted drug delivery in living organisms.

Multifunctional hybrid nanoparticles can further enhance 3D printing performance by synergistically combining the unique properties of inorganic and organic components. For example, Zhang et al. developed a 2D porphyrinic metal–organic framework (MOF) coated with polyvinylpyrrolidone, enabling dual functionality as a photocatalyst for SLA 3D printing and an antibacterial agent for photodynamic therapy.^[^ ^143^ ^]^ The porphyritic structure of 2D MOFs as photocatalysts to initiate polymerization obtained a nanocomposite with high resolution and well‐controlled structures in the 3D printing process.^[^ ^144^ ^]^ Under light irradiation, 2D MOFs can convert oxygen into singlet oxygen, which oxidizes proteins and lipids, leading to bacteria death. Antibacterial experiments displayed that 3D‐printed nanocomposites exposed to light have significant anti‐bacterial activity in gram‐positive and negative bacteria with 98% antibacterial effect on *S. aureus* and 93% antibacterial activity on *P. aeruginosa*. Overall, 3D‐printed composites for biomedical applications are still considered an emerging technology, primarily due to the high development costs and the need for long‐term biosafety evaluations. Thus, future research should prioritize the development of multifunctional nanocomposites that combine bioactivity, responsiveness, and improved printability to achieve higher precision and therapeutic efficacy, thereby increasing the potential for clinical translation. Overcoming these challenges will pave the way for next‐generation, personalized, and multifunctional composite biomedical devices.

### 3.2 Soft Robotics and Smart Materials

**Figure 5:**
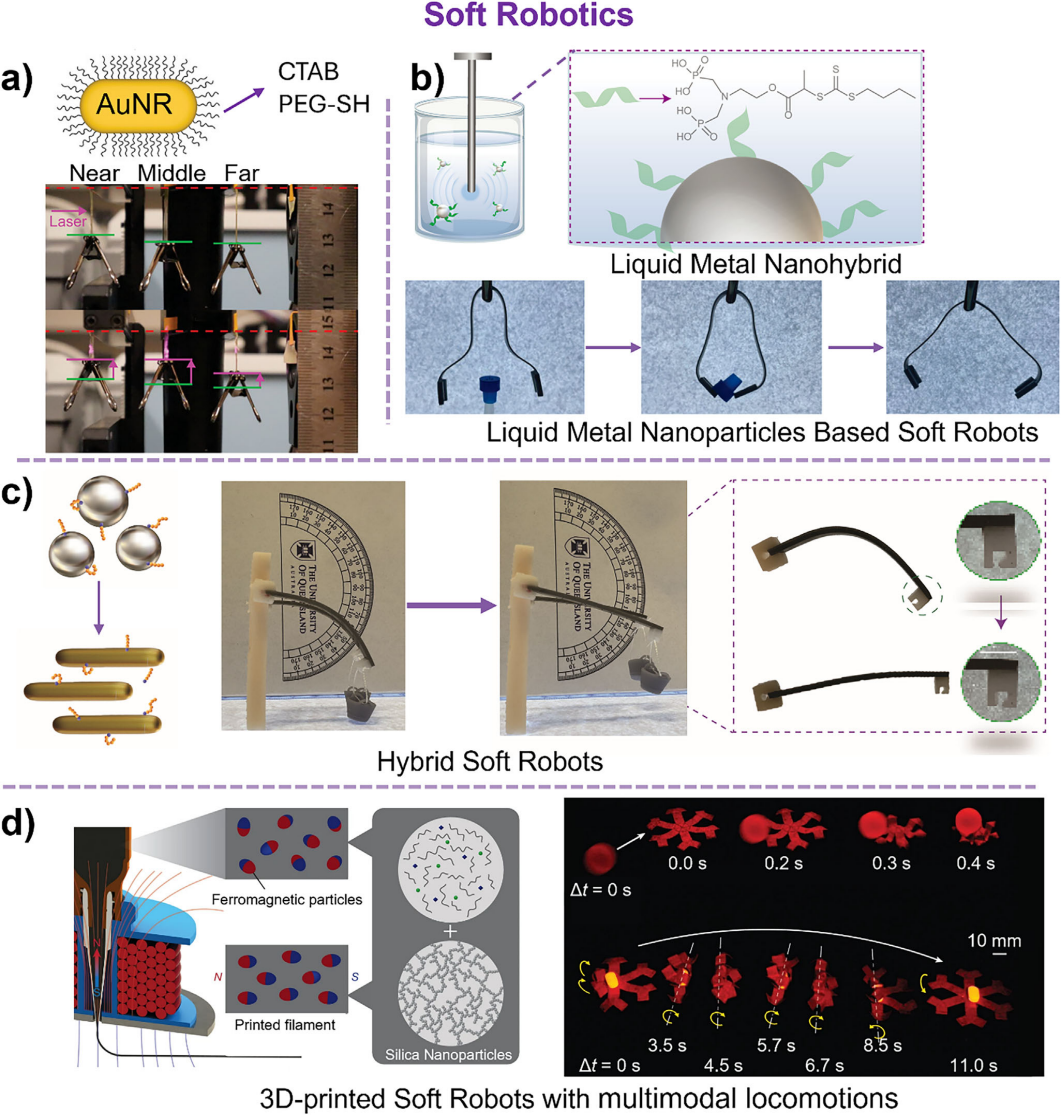
Innovations in 3D‐printed composites for soft robots. a). 3D‐printed liquid crystalline elastomer integrated with gold nanorods, showcasing efficient photothermal actuation. Reproduced with permission.^[^ ^110^ ^]^ Copyright 2024, Wiley‐VCH. b). 3D‐printed nanocomposites containing LMNPs, enabling near‐infrared (NIR)‐driven responsiveness in soft robots. Reproduced under the terms of the CC‐BY Creative Commons Attribution 4.0 International license (https://creativecommons.org/licenses/by/4.0).^[^ ^76^ ^]^ Copyright 2023, The Authors, published by Springer Nature. c). Hybrid nanocomposites created through 3D printing, offering advanced shape transformation for soft robotic systems. Reproduced under the terms of the CC‐BY Creative Commons Attribution 4.0 International license (https://creativecommons.org/licenses/by/4.0).^[^ ^71^ ^]^ Copyright 2024, The Authors, published by Wiley‐VCH. d). Ferromagnetic soft robots fabricated via 3D printing for multimodal locomotion. Reproduced with permission^[^ ^145^ ^]^ Copyright 2018, Springer Nature.

Soft robots are a class of robotic systems constructed primarily from flexible, compliant, and deformable materials, mimicking the adaptability and resilience found in natural organisms.^[^ ^146^ ^]^ Their unique flexibility enables them to carry out precise, and continuous movements, allowing for complex tasks such as crawling, rolling, running, and jumping.^[^ ^147^ ^]^ Soft robots have been employed in soft grippers, artificial muscles, wearables, medical devices, and environmental monitoring.^[^ ^148^ ^]^ Currently, the use of 3D‐printed nanocomposites in the development of advanced soft robots is also considered an emerging application. 3D printing has transformed the fabrication of soft robots by facilitating the creation of intricate geometries, high‐quality structures, and multilateral designs. The integration of hybrid nanoparticles has significantly enhanced the responsiveness of soft robots to external stimuli (e.g., light, electricity and heat), enhancing their capability to perform increasingly complex tasks with precision and efficiency. As shown in **Figure** **5**a, Skilin et al. employed a direct laser writing (DLW) printer to manufacture liquid crystalline elastomers (LCE)‐based composites containing 0.01 wt% PEG‐modified gold nanorods (PEG‐AuNR) as nanocomposites in 2024.^[^ ^110^ ^]^ LCEs, a class of advanced crosslinked polymers, exhibit permanent shape changes with heat and recover their original form upon cooling.^[^ ^149^ ^]^ PEG modification prevents AuNR aggregation and keeps uniform dispersion, which is beneficial to enhance photothermal efficiency. LCE‐AuNR nanocomposites, created via 3D printing, exhibited rapid photothermal heating rates of up to 150 °C s^−1^ under 808 nm NIR light (0.25 W cm^−^^2^), resulting in characteristic LCE directional contraction with rapid rates exceeding 60% s^−1^. NIR‐responsive contraction behavior showcased significant potential for next‐generation soft robotic systems.

4D printing is an advanced concept (3D printing + time) in additive manufacturing that enables static 3D‐printed objects to undergo shape transformations over time in response to external stimuli.^[^ ^150^ ^]^ It is worth noting that 4D printing effectively combines the precision and high resolution of 3D printing with the shape‐transforming capabilities of soft robotics, revolutionizing the field of robotics. The incorporation of hybrid nanoparticles allowing materials to withstand higher loads, resist impact, and endure fatigue into 3D‐printed structures offers considerable potential for enhancing the performance of 4D‐printed nanocomposites, as nanomaterials with distinctive properties, such as photosensitivity and chemosensitivity, can amplify the stimuli‐responsiveness of printed objects to initiate shape transformations. Although incorporating rigid nanoparticles into 4D printing enhances mechanical strength, it also reduces the softness and flexibility of soft robots, thereby limiting their shape‐transformation performance.

Unlike rigid nanoparticles, the soft and fluidic nature of gallium‐based liquid metals (LMs) can enable the fabrication of nanocomposites with softer mechanical properties while providing exceptional metallic properties, such as photochemical activity and high conductivity.^[^ ^151^ ^]^ Building on this concept, stereolithographic 3D printing was employed in our group to fabricate LMNPs‐based nanocomposites for photothermal‐responsive soft robotics.^[^ ^76^ ^]^ By successfully grafting chain transfer agents onto LMNPs, they synthesized hybrid nanoparticles with improved stability and dispersibility in various solvents, overcoming the limitations of bulk liquid metals. The resultant reactive LMNPs were directly incorporated into 3D‐printable resins, offering a simple and efficient one‐step printing method to produce nanocomposites (Figure 5b). The study demonstrated that incorporating LMNPs into 3D‐printed nanocomposites resulted in lower mechanical strength compared to those incorporating rigid Fe₃O₄ nanoparticles. Meanwhile, their softer and more fluidic nature made nanocomposites exhibit significantly faster shape transformations, with the programmed shape memory of the 3D‐printed composites returning to their original form within 60 s under NIR light irradiation. These nanocomposites were also successfully utilized as soft robots capable of grasping and releasing objects under NIR light irradiation, highlighting their potential for advanced applications in soft robotics.

Hybrid nanocomposites function as advanced systems that combine the properties of both soft and rigid components, enabling them to perform sophisticated tasks.^[^ ^152^ ^]^ However, existing fabrication techniques, including shape‐deposition manufacturing, 3D printing, laser cutting, lamination, and mold casting, often require intricate, multistep assembly, and time‐consuming processes are constrained by a limited range of compatible materials.^[^ ^153^ ^]^ Inspired by the shape‐transformable property of LMNPs, our group developed SLA‐printed hybrid robots via a direct one‐step 3D printing approach.^[^ ^71^ ^]^ LMNPs possess a unique shape transformation behavior, converting their morphologies and states of matter from spherical liquid nanoparticles to solid rods (Figure 5c).^[^ ^71^ ^]^ By integrating shape transformation capabilities into the 3D printing process, one‐step printing was achieved without the need for incorporating multiple types of nanoparticles. As shown in Figure 5c, the robot's upper limbs were constructed using LMNPs and nanorod‐based nanocomposites as different parts in hybrid robots. The arms, made from LMNPs composites with shape transformation ability, enable efficient item lifting under NIR light irradiation. In contrast, the hands, crafted from nanorod composites with high mechanical strength and minimal shape deformation, provided a firm grip on heavy objects, such as a bucket. Inspired by the flexion and extension of quadrupedal mammals’ spines, an innovative locomotive soft robot was manufactured by a one‐pot printing technique. In this design, LMNP‐based composites were regarded as the flexible functionality of muscles, while nanorod‐based composites provide sufficient structural support.

Research on soft robots is increasingly focused on achieving multimodal locomotion, which involves transitioning between various movement modes, such as crawling, walking, swimming, or climbing, to effectively navigate complex and dynamic environments.^[^ ^155^ ^]^ For example, magneto‐based robots exhibit remarkable versatility, capable of swimming both within and on the surface of liquids, climbing liquid menisci, rolling and walking on solid surfaces, jumping over obstacles, and crawling through narrow tunnels.^[^ ^156^ ^]^ In this case, magnetic nanoparticles are served as the key component for fabricating magneto robotics due to the reason that they not only exhibit exceptional dispersibility but also possess ferromagnetism, photothermal activity, and magnetocaloric effects, enabling them to respond effectively to multiple external photo and magnetic stimuli. Kim et al. employed the DLW method to fabricate soft materials with embedded ferromagnetic domains, enabling mutishape transformations, including reconfigurable soft electronics, a jumping mechanical metamaterial, and a multifunctional soft robot capable of crawling, rolling, catching fast‐moving objects, and transporting pharmaceutical doses.^[^ ^145^ ^]^ This advanced 3D printing technique significantly increased the complexity of soft robots. Taking a hexapedal structure as an example, the 3D‐printed hexapedal robot demonstrated the ability to stop and hold a fast‐moving object when exposed to a magnetic field generated by a permanent magnet. It could also wrap around and transport an oblong pharmaceutical pill using rolling‐based locomotion driven by a rotating magnetic field (Figure 5d). From 3D printing, hybrid robots to multimodal locomotion, the further development of soft robots is increasingly being applied to more and more practical scenarios.

### 3.3 Electronics

The scope of 3D‐printed nanocomposites has expanded into electronics, including fully 3D‐printed electronic devices, electronics containing 3D‐printed nanocomposites, and the direct fabrication of electrically conductive features on the surfaces of 3D‐printed nanocomposites, in which some products are already available in the market.^[^ ^161^ ^]^ 3D‐printed nanocomposites contribute to the electronics industry by enhancing material flexibility, reducing weight, and lowering costs, thereby unlocking new possibilities for diverse applications such as wearable electronics, flexible sensors, electromagnetic shielding, and energy devices. For the fabrication of 3D‐printed electronic devices, it is crucial to achieve specific properties such as electrical conductivity, dielectric behavior, magnetic response, and semiconductive functionality, as these characteristics directly influence device performance. AgNPs are among the most widely studied materials for creating highly conductive nanocomposites due to their exceptional electrical and thermal properties. They possess a high thermal conductivity of 427 W·m⁻¹·K⁻¹, an electrical conductivity of 62.9 × 10⁶ S·m⁻¹ at 293.15 K, and a melting temperature of 1234.93 K, making them ideal for applications requiring efficient charge transport and thermal dissipation.^[^ ^162^ ^]^

**Figure 6:**
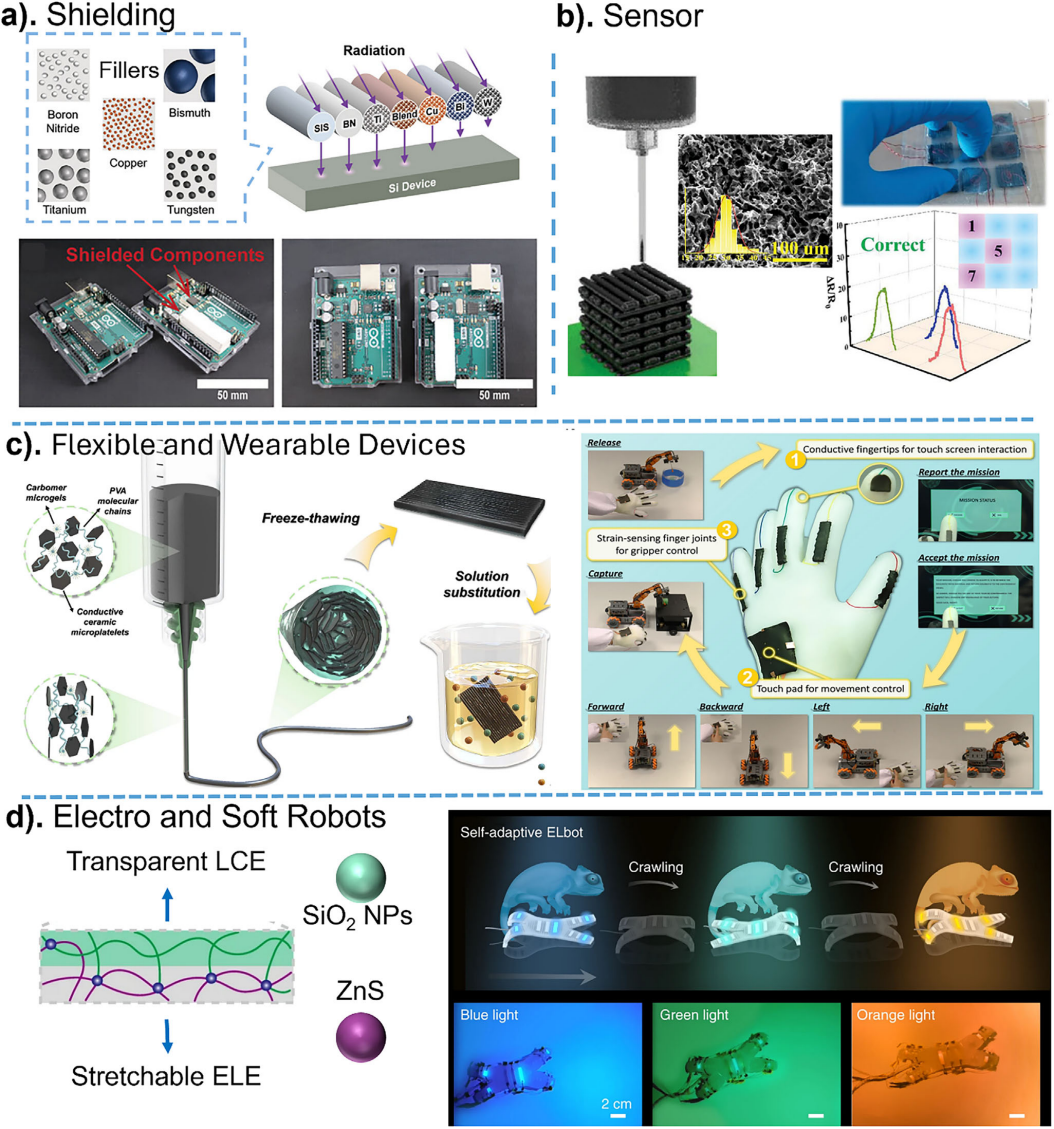
a). Integration of multiple techniques using fillers, binders, and surface modifiers with toluene for printing radiation‐shielding composites. Reproduced with permission^[^ ^157^ ^]^ Copyright 2024, Wiley‐VCH. b). 3D‐printed porous lattice sponge nanocomposites for skin‐inspired, flexible pressure sensors. Reproduced under the terms of the CC BY 4.0 license (https://creativecommons.org/licenses/by/4.0).^[^ ^158^ ^]^ Copyright 2024, The Authors, published by Wiley‐VCH. c). Schematic illustration of the fabrication process for composite organo‐hydrogel filaments via shear‐induced alignment in DIW 3D printing, demonstrating a multifunctional smart sensing glove with conductive fingertips, a touchpad, and strain sensors for touchscreen interaction and robotics control. Reproduced under the terms of the CC BY 4.0 license (https://creativecommons.org/licenses/by/4.0).^[^ ^159^ ^]^ Copyright 2024, The Authors, published by Springer Nature. d). Integration of 3D‐printed electronic devices into soft robots, enabling a chameleon‐inspired background‐matching strategy for Elbot. Reproduced under the terms of the CC BY 4.0 license (https://creativecommons.org/licenses/by/4.0).^[^ ^160^ ^]^ Copyright 2022, The Authors, published by Springer Nature.

However, the high cost of AgNPs has spurred extensive research into more cost‐effective alternatives, including other metal nanoparticles (e.g., copper, aluminum, and nickel) and nonmetallic materials such as graphene and CNTs for use in 3D‐printed electronics. Rosh‐Goryky utilized a DLW printer to create a versatile material platform that incorporates a flexible polymer binder with diverse nanofillers (e.g., boron nitride, titanium, bismuth, copper, etc.) for radiation shielding (**Figure** **6**a).^[^ ^157^ ^]^ The incorporation of particles with varying atomic densities within a single printed structure, this approach enables the fabrication of graded shielding through precise control over both the geometry and composition of the material. This design provides comprehensive protection against a broad spectrum of radiation species, including gamma rays, heavy ions, and high‐energy protons.

Sensors are essential electronic devices that detect environmental changes and transmit data for further processing.^[^ ^163^ ^]^ As the demand for miniaturized and highly precise sensors continues to grow—particularly in applications such as implantable monitoring devices, airbags, and other high‐precision instruments—3D printing has emerged as a promising fabrication method for sensors with micrometer‐ and millimeter‐scale structures. Liu et al. developed a DLW‐printed anisotropic piezoresistive pressure sensor by incorporating PEG‐modified single‐walled carbon nanotubes (SWCNTs) and cellulose nanofibrils into composites (Figure 6b).^[^ ^158^ ^]^ The sensor exhibited macroscopic 3D architectures with microscopic pore morphologies, providing exceptional compression resilience by returning to its original shape even after 50% strain. It also demonstrated increased resistance changes, significantly enhancing sensor sensitivity. When tested for robotic skin applications, the printed nanocomposites effectively detected a range of tactile interactions, including finger clicking, arm squeezing, cheek bulging, swallowing, and grasping/releasing motions. Notably, the sensor featured a novel password function, where resistance change signals were recorded based on specific pressure points, enabling unique user identification through tactile input. Additionally, Cui et al. developed polymer‐grafted zirconate titanate nanoparticle colloids, which were then shaped into complex 3D composites using high‐resolution additive manufacturing. These composites function as directional sensors, achieving a high piezoelectric charge and voltage constant even at low volume fractions while maintaining excellent flexibility.^[^ ^164^ ^]^

3D‐printed nanocomposites have also been applied to flexible and wearable devices for noninvasive interaction with the human body, which enables real‐time and continuous monitoring of physiological parameters for improved health management.^[^ ^165^ ^]^ 3D‐printed piezoelectric composites integrated into bioinspired structures enable the development of smart array armor and knee pads, leveraging 3D‐printed‐RSC (Rochelle salt cuttlebone composite) technology to accurately detect both the location and magnitude of forces experienced by the wearer.^[^ ^166^ ^]^ Yao et al. utilized a UV‐sensitive monomer matrix combined with lead zirconate titanate nanoparticles, a piezoelectric ceramic material, to fabricate highly sensitive, flexible, and customizable piezoelectric devices.^[^ ^167^ ^]^ Their work showcased the creation of robust and responsive 3D piezoelectric materials for wearable applications, capable of detecting subtle airflow pressures beyond the capabilities of commercially available piezoelectric polymers. They also developed wireless self‐sensing sporting gloves that integrate impact absorption with precise impact force mapping functionality.

One of the major challenges in 3D‐printed wearable devices is ensuring long‐term mechanical stability to withstand continuous wear and tear while maintaining durability and structural integrity over extended use. In 2024, Liu et al. applied hierarchical composite design principles inspired by natural materials to fabricate ceramic‐reinforced organo‐hydrogels using DLW printing (Figure 6c).^[^ ^159^ ^]^ These materials demonstrate exceptional mechanical properties, including high stiffness, strength, and toughness, with fracture energy reaching up to 31.1 kJ m^−2^, achieved through synergistic multiscale energy dissipation mechanisms. The hydrogels exhibit excellent electrical conductivity and broad operational tolerance, enabling their use in a multifunctional smart sensing glove with features like touch screen interaction, robotic vehicle control, and gripper operation via strain sensors.

Another emerging strategy involves integrating wearable devices into diverse fields. A DLW printer was employed to fabricate 3D‐layered composites, including ion‐conducting elastomers with SiO₂ nanoparticles, electroluminescent elastomers embedded with ZnS phosphor microparticles, and insulating dielectric elastomer layers (Figure 6d).^[^ ^160^ ^]^ These composites exhibited programmable spatial color‐matching capabilities, allowing each pixel to dynamically adjust based on local environmental stimuli. Notably, these devices were successfully integrated into soft robots, enabling autonomous natural camouflage by detecting background colors and dynamically modifying surface coloration to seamlessly blend into the environment. This advancement highlights the potential of 3D‐printed nanocomposites in adaptive smart materials, bio‐inspired robotics, and next‐generation wearable electronics.

It is well recognized that DLW printing technology is primarily employed in the fabrication of electronic components. DLW systems are inherently costly, requiring specialized laser sources, advanced optics, and precision motion control systems. As a point‐by‐point writing process, DLW has a relatively slow printing speed compared to other additive manufacturing techniques, limiting its suitability for large‐scale production. Therefore, exploring alternative 3D printing methods is crucial for advancing electronic device fabrication.

### 3.4 Water Treatment

Currently, environmental pollution, like water and air pollution, represents a significant threat to both human health and biodiversity. Wastewater treatment is the process of removing contaminants from used water through physical, chemical, and biological methods to make it safe for discharge or reuse.^[^ ^172^ ^]^ Traditional wastewater treatment methods, such as thermal treatment methods, activated carbon adsorption, chemical coagulation and flocculation, are designed to remove contaminants and improve water quality.^[^ ^173^ ^]^ In the era of Industry 4.0, advanced 3D printing techniques offer a highly promising and efficient solution for wastewater treatment, facilitating the rapid product commercialization.^[^ ^174^ ^]^ 3D printing enables the rapid and cost‐effective fabrication of customized geometric structures while minimizing waste. This technology enhances the functionality of fabricated materials or provides additional functions, enabling efficient pollutant removal through mechanisms such as photocatalysis, separation, and adsorption. It has been applied in various water treatment scenarios, including filtration and desalination membranes, oil‐water separation, heavy metal, and organic removal.

**Figure 7:**
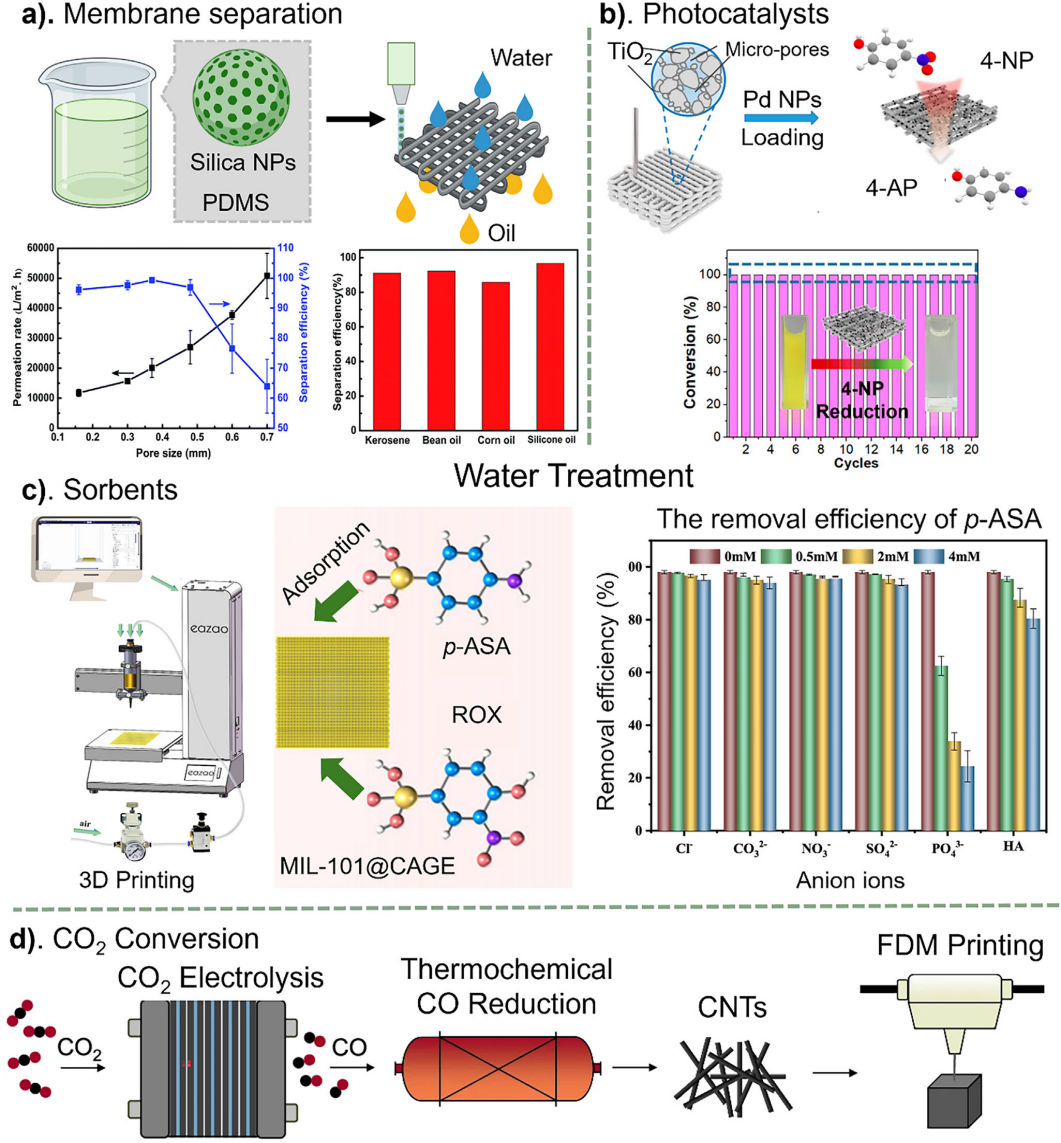
3D‐printed nanocomposites for wastewater treatment. a). 3D‐printed hydrophobic nano silica‐filled membranes with an ordered porous structure for oil‐water separation. Reproduced with permission.^[^ ^168^ ^]^ Copyright 2017, Royal Society of Chemistry. b). 3D‐printed Pd/TiO_2_ nanocomposites tailored for the reduction of high concentrations of 4‐nitrophenol in aqueous environments. Reproduced with permission.^[^ ^169^ ^]^ Copyright 2020. American Chemical Society. c). 3D‐printed MIL‐101/Polymer composite is regarded as a reusable adsorbent for the removal of phenyl arsenic acid from water solutions. Reproduced with permission.^[^ ^170^ ^]^ Copyright 2023, American Chemical Society. d). A schematic diagram illustrates the conversion of CO_2_ into CNTs for printing carbon‐based nanocomposites. Reproduced under the terms of the CC BY 4.0 license (https://creativecommons.org/licenses/by/4.0).^[^ ^171^ ^]^ Copyright 2024, The Authors, published by Springer Nature.

Membrane is widely used in water treatment for their high efficiency and cost‐effectiveness, but conventional approaches, e.g. phase inversion, hollow fiber spinning, solvent casting, and extrusion, require precise parameter control, making the process complex and time‐consuming.^[^ ^175^ ^]^ Conventional membrane fabrication methods often produce structures with limited flexibility, restricting their applicability in advanced or specialized filtration systems. In contrast, 3D printing techniques enable the fabrication of membranes with superior design flexibility, high precision, and fine‐tuned control over structural thickness and porosity, making them increasingly valuable for water filtration and oil‐water separation. Chen et al. reported 3D‐printed superhydrophilic ceramic meshes embedded with in situ grown aluminum borate whiskers, achieving over 99% efficiency in oil/water separation.^[^ ^176^ ^]^ Furthermore, some researchers are actively exploring next‐generation 3D‐printed membranes.^[^ ^177^ ^]^ Lv et al. developed a custom 3D printing method to create superhydrophobic membranes with an ordered porous structure for oil‐water separation, using hydrophobic nanosilica‐filled polydimethylsiloxane ink (**Figure** **7**a).^[^ ^168^ ^]^ The incorporation of high‐surface‐area nanosilica maintained the ink stability and prevented collapse during the printing process. Interestingly, the pore size of 3D‐printed membranes could be precisely customized, enabling optimization parameters like liquid flux and membrane separation efficiency. The resulting 3D‐printed membrane, with a 0.37 mm pore size, achieved an impressive oil‐water separation efficiency of 99.6% and a high liquid flux of 23700 L·m^−2^·h^−1^, demonstrating its potential for advanced wastewater treatment applications.

Photocatalysts have proven to be one of the highly effective approaches for wastewater treatment.^[^ ^178^ ^]^ These photocatalysts react with dissolved oxygen in water under specific light irradiation, generating ROS, like hydroxyl radicals (·OH) and superoxide radicals (O^2−^). These highly reactive species effectively degrade organic pollutants, pesticides, and dyes, breaking them down into harmless byproducts, making photocatalysis a powerful approach for wastewater treatment.^[^ ^179^ ^]^ To date, some 3D printing techniques, including material jetting, binder jetting, material extrusion, FDM, and vat photopolymerization, have been employed to manufacture nanocomposites containing heterogeneous photocatalyst nanoparticles for treating wastewater.^[^ ^180^ ^]^ ZnO nanoparticles, surface‐coated with PLA, were integrated into FDM printing to fabricate 3D‐printed nanocomposites, achieving a 63% degradation efficiency for Rhodamine B—a toxic, carcinogenic, and nonbiodegradable water pollutant.^[^ ^181^ ^]^

Similarly, SLA‐printed nanocomposites embedded with titania nanoparticles in a silver matrix demonstrated great photodegradation of Rhodamine B and achieved 89% antibacterial efficiency utilizing photocatalytic activity.^[^ ^180^ ^]^ Among these photocatalysts, TiO₂ NPs are the most widely used in 3D printing techniques due to their exceptional electronic and optical properties, making them highly effective in generating ROS under UV light to degrade organic pollutants. Liu et al. printed Pd/TiO_2_ hybrid composites by uniformly coating palladium (Pd) nanoparticles onto a porous TiO_2_ scaffold (Figure 7b).^[^ ^169^ ^]^ The synergistic interaction between the TiO_2_ and Pd nanoparticles significantly enhanced the catalytic performance,^[^ ^182^ ^]^ efficiently reducing high concentrations of 4‐nitrophenol (4‐NP) in wastewater (2 g·L^−1^, ≈14.38 mm) with no significant loss in activity even after 20 consecutive cycles. Additionally, TiO_2_‐based nanocomposites were utilized to degrade polycyclic aromatic hydrocarbons in water under UV light irradiation.^[^ ^183^ ^]^ It is worth noting that poor light penetration of nanoparticles limited the photocatalytic efficiency of 3D‐printed nanocomposites in wastewater treatment.

The adsorption of organic contaminants is a cost‐effective and readily accessible strategy for wastewater treatment. The adsorption‐based method employs sorbents that can selectively capture specific contaminants like molecules and ions. 3D‐printed nanocomposites as excellent sorbent exhibits superior mechanical strength, controllable porosity, high stability, and efficiency. For example, MOFs have been successfully integrated with 3D printing to manufacture sorbent nanocomposites for the removal of organic dyes in wastewater.^[^ ^184^ ^]^ Their extensive surface area provides a high adsorption capacity, while abundant metal active sites facilitate strong interactions with dye molecules, and their highly porous structures enhance mass transfer and adsorption kinetics, collectively leading to efficient pollutant removal.

Liu et al. employed DLW techniques to prepare Cu‐MOF‐based nanocomposites, demonstrating high adsorption efficiency by achieving 90% removal of malachite green within just 10 min.^[^ ^185^ ^]^ Among all MOFs, MIL‐101, with its smallest pore size and remarkable flexibility, has become an ideal candidate for the adsorption of toxic substances in water. Li et al. utilized an SLS printer to fabricate MIL‐101‐polymer nanocomposites, effectively absorbing 152 mg g^−1^ of methylene blue from water while maintaining a removal efficiency of 81.3% after five cycles.^[^ ^186^ ^]^ Furthermore, Ding et al. developed a hybrid MIL‐101@CAGE nanocomposite by mixing MIL‐101 (Fe), sodium alginate, and gelatin in 2023 (Figure 7c).^[^ ^170^ ^]^ This composite was designed as a separable adsorbent for the removal of phenylarsenic acid from aqueous solutions through multiple interactions, including *π–π* stacking interactions, hydrogen bonding, and ligand‐bonding interactions. Results showed that the maximum adsorption capacities of MIL‐101@CAGE for *p*‐arsanilic acid and roxarsone at 25 °C were 106.98 and 120.28 mg g^−1^, respectively. Notably, the 3D‐printed MIL‐101@CAGE nanocomposites maintained over 90% removal efficiency even after five consecutive cycles, highlighting their durability and reusability for wastewater treatment applications.

Beyond wastewater treatment, 3D printing techniques are also utilized for environmental pollution control, particularly in the fabrication of advanced nanocomposites designed for efficient air pollutant removal. The rising concentration of atmospheric carbon dioxide (CO_2_) is a pressing global issue that profoundly impacts climate patterns and Earth's ecological balance.^[^ ^187^ ^]^ 3D printing techniques enable the rapid prototyping of electrodes and devices tailored for CO_2_ reduction.^[^ ^188^ ^]^ Thakkar et al. demonstrated two FDM‐printed nanocomposites containing 80 and 85 wt% of MOF‐74(Ni) and UTSA‐16(Co) for gas capture.^[^ ^189^ ^]^ Experimental results revealed that when exposed to 5000 ppm (0.5%) CO₂ at 25 °C, the MOF‐74(Ni) and UTSA‐16(Co) based nanocomposites exhibited CO₂ adsorption capacities of 1.35 and 1.31 mmol g^−1^, respectively. However, CO₂ capture and storage (CCS) remains a subject of concern due to potential long‐term environmental risks, regulatory challenges, and the possibility of leakage, which may undermine its effectiveness as a sustainable solution.

As an alternative, electrocatalytic CO₂ conversion has gained interest, as it facilitates multiproton and multielectron transfer reactions, yielding a range of valuable chemical products.^[^ ^190^ ^]^ including carbon monoxide (syngas production; CO+H_2_), formic acid (HCOOH), alcohols (methanol, ethanol, etc.), hydrocarbons (e.g., methane, ethane, ethylene, etc.), aldehyde, ketone, etc. Crandall et al. utilized an FDM printer to fabricate nanocomposites capable of converting CO₂ into CNTs in November 2024.^[^ ^171^ ^]^ As shown in Figure 7d, CO_2_ was first electrochemically reduced to CO under ambient conditions, followed by CO dissociation on a catalyst surface in a thermochemical reactor, facilitating the formation of CNTs. This innovative approach reduces CNT production costs by 90% compared to the conventional fossil fuel‐based method. Nanocomposites incorporating ≈38 wt% of these CO_2_‐derived CNTs were successfully 3D‐printed, demonstrating a Young's modulus of 1.61 GPa and 87% higher thermal conductivity. While the proposed strategy is innovative, this two‐step process—first converting CO₂ into CNTs and then integrating the nanotubes into 3D printing—introduces additional complexity. In the future, it would be advantageous to develop a more convenient approach that directly employs 3D‐printed nanocomposites capable of converting polluting gases into chemicals or fuels.

Despite significant advancements, scaling up the production of 3D‐printed nanocomposites remains a critical challenge, particularly for wastewater treatment applications, where large‐scale implementation is essential for practical and industrial viability.

### 3.5 Other Applications

3D‐printed nanocomposites hold immense potential across a wide range of industries. In recent years, the aerospace industry has increasingly focused on materials that offer a balance of low weight, superior mechanical strength, thermal stability, and wear resistance.^[^ ^191^ ^]^ Conventional materials often struggle to meet these requirements, prompting a shift toward advanced nanocomposites. By incorporating hybrid nanoparticles, these composites enhance mechanical strength, thermal conductivity, resistance, and corrosion protection, while simultaneously reducing overall weight. Carbon‐based nanoparticles, such as graphene and CNTs, are widely used to improve the performance of aerospace materials. Graphene, which is lighter yet stronger than steel, also exhibits exceptional flexibility and can significantly enhance thermal and electrical conductivity.^[^ ^192^ ^]^ Similarly, CNTs are widely used to produce high‐performance polymer composites due to their excellent flexibility, low density, and remarkable strength.

Currently, polymer composites account for ≈50% of the materials used in aircraft structural components.^[^ ^193^ ^]^ The Boeing 787 marked a significant milestone as the first aircraft to extensively utilize composites as primary materials, particularly in the construction of its main wings and fuselage. In modern aircraft, composites now constitute nearly half of the total weight, reflecting their crucial role in reducing weight and enhancing performance. The emergence of 3D printing technology has further transformed aerospace manufacturing and enabled the production of lightweight, complex geometries while reducing material waste and energy consumption. This additive manufacturing approach minimizes the material required for aircraft components, contributing to fuel savings and operational efficiency. Consequently, 3D‐printed carbon‐polymer nanocomposites represent the future of aerospace manufacturing, offering a scalable and cost‐effective solution for next‐generation aviation materials.

Similarly, in energy‐related applications, 3D‐printed nanocomposites have been used in energy generation, conversion, and storage.^[^ ^194^ ^]^ Although the electrical output of 3D‐printed energy devices may not always surpass that of conventionally manufactured counterparts, 3D‐printed nanocomposites offer unique advantages, including customizability, material diversity, process flexibility, and precise geometric control. These factors make it a promising platform for energy device fabrication. 3D‐printed composites, particularly micro‐lattice structures, demonstrate superior mechanical and electrical properties compared to their bulk counterparts. By integrating nanoparticles, these composites offer enhanced conductivity, superior mechanical strength, and multifunctionality. Maurya et al. employed a DLP printer to make the lattice composites containing 10 wt% ZnO, achieving the maximum power density of 0.065 µW cm^−2^ under 12 N force at 1 Hz in 2024.^[^ ^195^ ^]^ Similarly, Zhang et al. used a multinozzle FDM 3D printer to develop nano‐diamond/ZnO nanocomposites for solar cells.^[^ ^196^ ^]^

In addition to aerospace and energy, 3D‐printed nanocomposites are driving innovation in food packaging, architecture, and construction. In the food industry, these materials are used to create high‐performance packaging with enhanced barrier properties, improving food preservation and reducing waste. In construction and architecture, 3D‐printed nanocomposites enable the fabrication of complex, sustainable structures with improved mechanical properties, thermal insulation, and lower environmental impact. In conclusion, 3D‐printed nanocomposites are revolutionizing medical devices, soft robots, wastewater treatment, aerospace, energy, biomedical, environmental, and industrial applications, paving the way for next‐generation materials that combine precision, functionality, and sustainability in modern manufacturing.

## 4 Challenges and Perspectives

### 4.1 Current Challenges

**Figure 8:**
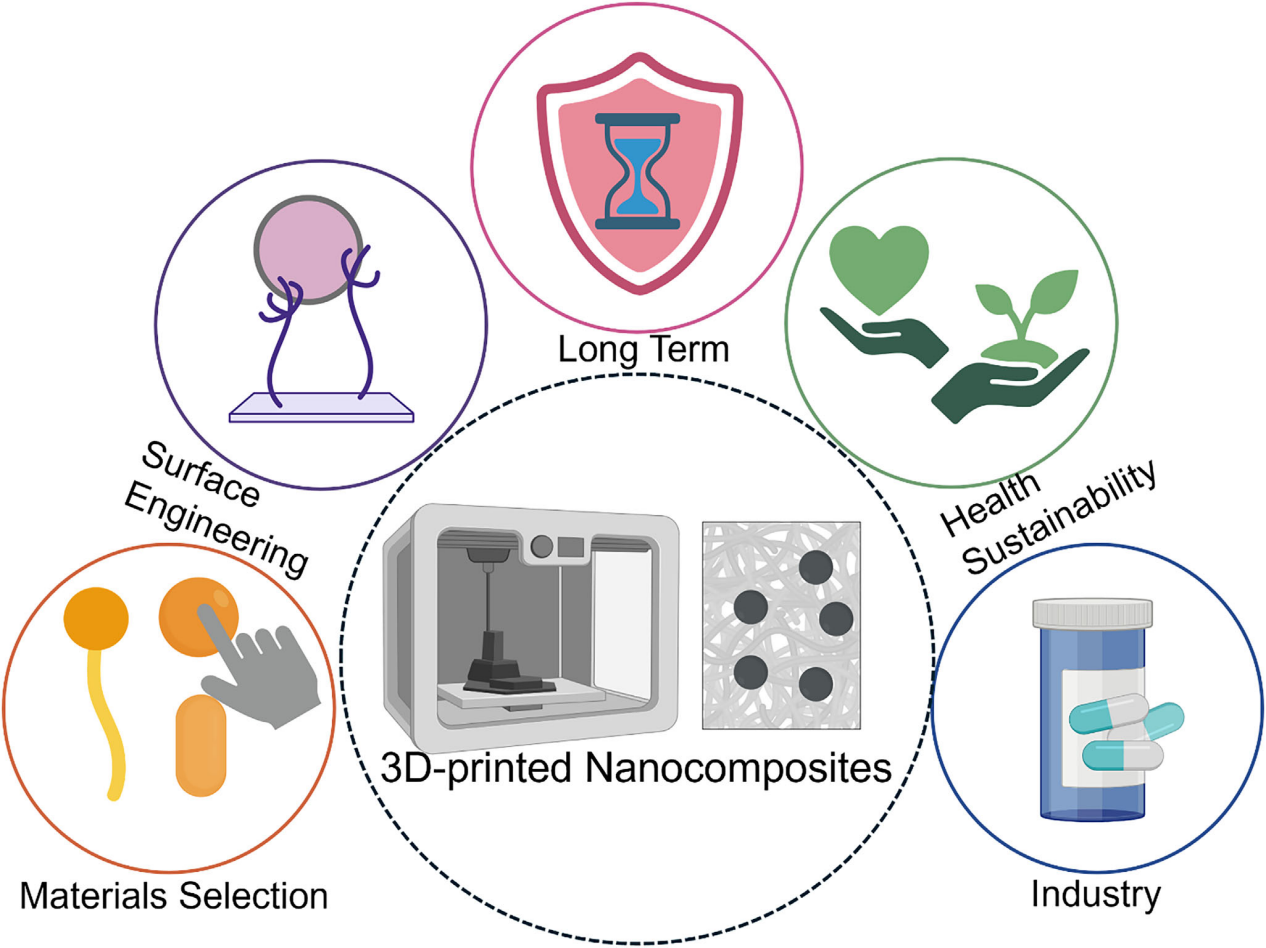
Schematic illustration highlights the main challenges in 3D‐printed nanocomposites containing hybrid nanoparticles. These challenges include material selection, hybrid nanoparticle‐matrix interactions, long‐term durability, health and sustainability concerns, and industrial scalability. Created using BioRender.com.

Although rapid advancements in 3D printing and nanocomposites have transformed various fields, such as healthcare, soft robotics, electronics, and environmental sustainability, several key challenges remain in 3D‐printed nanocomposites, including material selection, nanoparticle‐matrix compatibility, long‐term stability, environmental and health impacts, and industrial scalability (**Figure** **8**).

#### 4.1.1 Materials Selection

Inappropriate selection of materials limits the performance, structural fidelity, and functions of 3D‐printed nanocomposites. High loading greatly increases the viscosity of photopolymer resins, impairing their ability to flow and spread uniformly during printing. The aggregation and high concentrations of nanoparticles cause the random scattering of UV light in vat polymerization resins,^[^ ^197^ ^]^ reducing UV penetration depth and compromising lateral resolution, which ultimately affects the precision and quality of printed structures. High concentrations also raises the viscosity of photosensitive resins, which in turn reduces the polymer self‐diffusion coefficient.^[^ ^198^ ^]^ This reduction negatively impacts the resin's leveling properties, resulting in poor surface quality of printed structures.

Therefore, the selection of appropriate materials is critical for improving the properties and overall performance of 3D‐printed nanocomposites, as it relies on the rational alignment of characteristics with the specific processing requirements of each printing technique. As summarized in **Table** **3**, vat photopolymerization like SLA and DLP demands the use of nanofillers (e.g., silica nanoparticles and QDs) that exhibit low optical scattering and minimal UV absorption, ensuring uniform light penetration, effective curing, and high‐resolution feature fidelity. Extrusion‐based techniques like FDM and DIW require hybrid nanoparticles with high thermal stability and well‐controlled viscosity, as poor dispersion or excessive loading can lead to nozzle clogging and void formation during extrusion. Meanwhile, inkjet‐based methods impose strict constraints on both viscosity and particle size, necessitating the use of sub‐100 nm nanoparticles in low‐viscosity resins to ensure stable droplet formation, prevent nozzle clogging, and achieve precise deposition. Overall, each 3D printing technique has unique constraints on physicochemical properties, and the use of inappropriate materials can significantly compromise print quality, structural integrity, and functional performance.

**Table 3:** Design guidelines for selecting appropriate materials for 3D printing.

3D printing technique	Material requirements	Challenges	Design recommendations
Vat photopolymerization	Low UV absorption/scattering, high dispersion stability, and minimal viscosity increase	Light scattering, phase separation, and poor curing depth	Surface‐functionalized nanoparticles, optimize loading, match refractive index with the resin
Extrusion‐based printing	Thermal stability, good compatibility, and low aggregation tendency	Nozzle clogging, void formation, and poor filler dispersion	Limit loading to maintain flowability, functionalize surfaces
Inkjet and jet printing	Low viscosity, stable suspensions, and small particle size	Nozzle clogging, coffee‐ring effect, and poor adhesion	Use dispersants/surfactants, modify ink rheology
Direct ink writing	Shear‐thinning behavior, gelation, or self‐support	Collapse after extrusion, inhomogeneous deposition	Control yield stress and viscoelasticity

#### 4.1.2 Surface Modification

Surface coating is an effective strategy for improving the dispersity and stability of nanoparticles and plays a crucial role in facilitating stronger interfacial interactions between nanoparticles and the polymer matrix through van der Waals forces, hydrogen bonding, and chemical reactions. These interactions improve load transfer, mechanical integrity, and overall composite performance, optimizing the properties of 3D‐printed nanocomposites. However, several limitations must be addressed to ensure long‐term stability and enough functionality of hybrid nanoparticles in 3D‐printed nanocomposites. In particular, weak intermolecular forces such as van der Waals interactions and electrostatic attraction in surface engineering techniques may not provide sufficient long‐term stability. Over time, these weak interactions can lead to polymer detachment.

Physical coating is a widely used technique for fabricating 3D‐printed nanocomposites; however, it often lacks mechanical robustness. The electrostatic interactions between hybrid nanoparticles and 3D‐printed structures are inherently weak, making them susceptible to delamination or poor adhesion under mechanical stress or environmental exposure. Additionally, some surface engineering strategies relying on chemical coupling are sensitive to external factors such as UV exposure, high temperatures, enzymatic activity, and protein interactions, which can accelerate nanocomposite degradation. For 3D‐printed nanocomposites intended for long‐term applications in demanding environments—such as heart valves, implants, and electronic components—ensuring long‐term stability is critical. Therefore, careful evaluation of their durability and resistance to environmental stressors must be prioritized in the design and material selection process.

#### 4.1.3 Long‐Term Durability

3D‐printed nanocomposites need long‐term durability, particularly in applications requiring prolonged mechanical stability, environmental resistance, and structural integrity. This is especially critical in fields such as medical devices, soft robotics, and wearable electronics, where materials must endure prolonged mechanical stress and external environmental factors without significant degradation. One of the primary challenges to durability is cyclic stress, which can induce fatigue‐related microcracks and ultimately lead to structural failure. The incorporation of hybrid nanoparticles further reduces durability by generating potential voids within the polymer matrix and weak interfacial interactions. These deficiencies make the material more susceptible to microcrack initiation and propagation under mechanical stress. Additionally, poor dispersion tends to reaggregate over time, creating localized stress concentration points that further weaken the composite and accelerate mechanical degradation. Addressing these issues requires improved nanoparticle dispersion, optimized interfacial bonding strategies, and advanced post‐processing techniques to enhance the long‐term performance and reliability of 3D‐printed nanocomposites.

#### 4.1.4 Health and Sustainability Concerns

The integration of hybrid nanoparticles into 3D‐printed materials raises significant health and sustainability concerns, particularly in biomedical applications. Their nanoscale dimensions make them penetrate human cell membranes, posing potential risks, such as DNA damage, immune system disruption, and organ accumulation. Metal‐based nanoparticles (e.g., Ag, TiO_2_, and ZnO) and carbon‐based nanoparticles (e.g., CNTs and graphene) have demonstrated varying degrees of toxicity. These risks are especially critical for 3D‐printed wearable and implantable devices, where prolonged exposure to nanomaterials may lead to unforeseen biological interactions.

3D‐printed nanocomposites face several issues with environmental sustainability. While using 3D‐printed nanocomposites is an effective method in wastewater treatment by removing contaminants, they may also inadvertently release hybrid nanoparticles from 3D‐printed nanocomposites into the environment. As degradable nanocomposites break down, there is a potential risk that the uncontrolled release into the environment may lead to bioaccumulation, toxicity to marine organisms, and potential long‐term ecological disruptions. Therefore, ensuring that both the polymer matrix and nanoparticles degrade into nontoxic, environmentally benign byproducts is crucial for minimizing adverse effects and promoting sustainable applications of 3D‐printed nanocomposites.

#### 4.1.5 Industrial‐Scale Production

3D‐printed nanocomposites face one challenge in industrial‐scale production. Over the years, traditional manufacturing has become increasingly efficient, accurate, and reliable. In contrast, 3D printing remains considerably slower, making it less viable for high‐volume manufacturing. For instance, while a 3D printer requires ≈1 h to produce a 38 mm cube, injection molding can fabricate multiple similar cubes within a minute.^[^ ^199^ ^]^ This stark difference in production speed highlights the scalability limitations of 3D printing for high‐volume manufacturing. The integration of hybrid nanoparticles into 3D printing systems further imposes limitations on fabrication speed and resolution. In extrusion‐based systems, hybrid nanoparticles often increase viscosity and change the rheological behavior of printing inks, thereby complicating material extrusion and requiring slower deposition rates to maintain print fidelity. The agglomeration of hybrid nanoparticles can obstruct nozzles and cause inconsistent flow, further degrading resolution and structural precision. In vat photopolymerization, the presence of hybrid nanoparticles can interfere with light penetration and curing efficiency by scattering or absorbing the projected light, thereby reducing resolution and curing depth. Furthermore, the agglomeration can lead to inhomogeneous material distribution and inconsistent mechanical or functional properties. To overcome these limitations, recent research and technological innovations have targeted improvements in printing resolution and fabrication speed for nanocomposite‐based 3D printing. One approach involves the development of low‐viscosity inks to enhance dispersion and reduce nozzle clogging, thereby improving flow behavior and maintaining print resolution even at higher speeds.^[^ ^200^ ^]^ Advanced photopolymer formulations have been engineered to minimize light scattering in vat photopolymerization systems, allowing for deeper and more uniform curing.^[^ ^93^ ^]^ Additionally, multinozzle parallel printing and continuous 3D printing systems, such as Continuous Liquid Interface Production (CLIP), have emerged as promising approaches to significantly enhance fabrication speed without compromising structural integrity. The multinozzle parallel printing technique employs multiple print heads or nozzles operating simultaneously, with each responsible for a designated portion of a single object or for printing multiple objects.^[^ ^201^ ^]^ This parallelization strategy substantially reduces the overall production time, particularly in large‐scale or high‐throughput manufacturing processes. CLIP represents a novel vat photopolymerization process that enables continuous object manufacturing rather than the traditional layer‐by‐layer approach.^[^ ^202^ ^]^ It achieves printing speeds up to 25–100 times faster than conventional SLA and DLP, while also providing enhanced surface finish and more isotropic mechanical properties, making it a compelling candidate for industrial‐scale 3D printing applications.

The high cost of advanced 3D printing equipment, particularly metal 3D printers, presents a significant financial barrier to widespread industrial adoption. Industrial‐grade 3D metal printers, such as those utilizing selective laser melting (SLM) or electron beam melting (EBM), can range in price from tens of thousands to several hundred thousand dollars. In addition to the initial investment, operational expenses, including maintenance, specialized powder materials, and post‐processing requirements—substantially increase the total cost of ownership. These financial constraints limit the accessibility of 3D printers, particularly for small and medium‐sized businesses. Currently, its adoption is primarily restricted to high‐value applications where the benefits of 3D printing outweigh the costs, such as aerospace, medical implants, and specialized automotive components. One strategy to reduce the financial barriers of industrial 3D printing involves the selection of cost‐effective printing technologies, which can better suit specific applications. For instance, binder jetting offers more economical alternatives to SLM for certain metal parts. Additionally, the use of recycled materials, open‐source platforms, and shared manufacturing services has improved accessibility for small and medium‐sized enterprises, gradually expanding industrial adoption beyond high‐value sectors.

### 4.2 Perspective

Despite the challenges, the combination of hybrid nanoparticles and 3D printing remains at the forefront of innovation in advanced material design and functionality. At the same time, it is unlocking new possibilities through the introduction of cutting‐edge techniques, including:

#### 4.2.1 5D Printing

Although the concept of 5D printing has been proposed, a unified definition has yet to be established. In the view of the printer, 5D printing incorporates two additional axes, involving the movement of the printing head and the print bed at a specific angle.^[^ ^203^ ^]^ Embedded information—such as functional nanoparticles, genes, growth factors, and cellular signals—serves as the fifth dimension alongside 3D space and time in 5D printing.^[^ ^204^ ^]^ This integrated information enables targeted biological or structural transformations in 5D‐printed structures while retaining the main capabilities of 3D and 4D printing. For example, advanced tissue engineering products can be created that integrate shape transformation capabilities with the controlled delivery of embedded information in 5D printing. These products can dynamically change shape to adapt to their environment while releasing molecular signals to promote cell differentiation and growth, facilitating the regeneration of complex body tissues.

**Figure 9:**
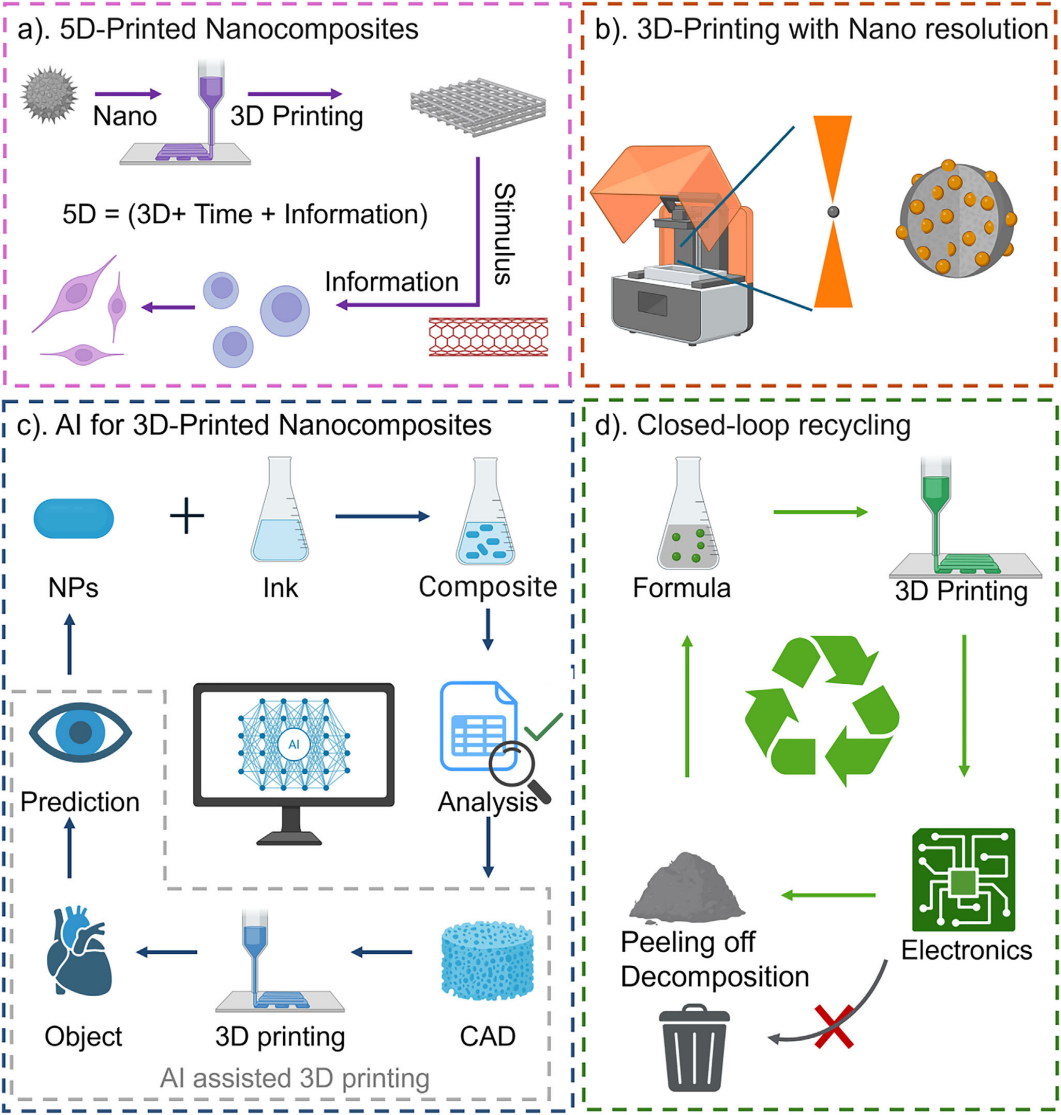
The perspectives of 3D‐printed nanocomposites with hybrid nanoparticles. a) 5D‐printed nanocomposites, integrating 3D printing with time‐dependent shape transformation and embedded information; b) Emerging techniques for achieving nanoscale resolution in 3D‐printed objects; c) Artificial intelligence (AI)‐driven advancements for optimizing 3D printing and nanocomposite performance; d) Closed‐loop recycling enabled by 3D‐printed nanocomposites. Created with BioRender.com.

Hybrid nanoparticles serve as a versatile platform for 3D, 4D, and 5D printing. Functional hybrid nanoparticles enhance the strength, durability, chemical and electrostatic properties of 3D‐printed nanocomposites while also offering external stimuli responsiveness to initiate shape‐transformation of 4D printing. More importantly, hybrid nanoparticles could act as embedded information, dynamically controlling the mechanical properties and biochemical cues of 5D‐printed nanocomposites to promote cell growth and differentiation for tissue engineering and regenerative medicine (**Figure** **9**a). Although 5D printing is still in its infancy, the incorporation of hybrid nanoparticles is expected to accelerate its development and expand its potential applications.

#### 4.2.2 Nanoscale 3D Printing Techniques

High‐precision industries, such as semiconductor chip manufacturing, nano‐optical devices, and microfluidics, not only require highly complex structures but also demand exceptional resolution at the micron or even nanometer scale to ensure design accuracy and final performance. Microfabrication techniques have demonstrated the ability to produce structures across micro‐ and macro‐scales due to their high resolution and excellent surface finish.^[^ ^205^ ^]^ Among the emerging methods, micro‐digital light processing and TPP 3D photolithography achieve the fabrication of structures with feature sizes below 10 µm. The micro‐DLP method is able to print 3D structures with feature sizes at the 100‐µm scale.^[^ ^206^ ^]^ Meanwhile, TPP has been successfully applied in fabricating microfluidic channels, microneedles, and microswimmers, establishing itself as a powerful technique for applications in lab‐on‐a‐chip systems, biomedical diagnostics, and chemical analysis platforms.^[^ ^207^ ^]^

As advancements in science and technology accelerate, nanomanufacturing technologies—defined as the fabrication of structures and devices with features typically below 100 nm—has gained growing attention as a platform for realizing next‐generation functional materials. In contrast to microfabrication, nanofabrication offers atomic‐ and molecular‐level control over structural and functional properties, a capability that is essential for applications in quantum computing, nanophotonics, and nanoscale biomedical systems. For example, Han et al. utilized ultrafast laser patterning (>1 TW cm^−2^) to manufacture nanodevices like encrypted optical storage and microelectrodes. This approach allowed precise control over the formation of high‐index nanophotonic components with feature sizes below 50 nm, addressing key challenges in integrating photonic structures into next‐generation chips.^[^ ^208^ ^]^ Furthermore, recent innovations in high‐resolution laser‐based nanofabrication have significantly expanded the scope of 3D printing at the nanoscale. Xie et al. fabricated high‐resolution 3D silica structures by formulating a specialized ink composed of a two‐photon polymerizable precursor and PEG‐functionalized colloidal silica nanoparticles (<10 nm, Figure 9b) using the TPP 3D printing approach.^[^ ^209^ ^]^ This method enabled the creation of high‐quality 3D architectures with arbitrary geometries in either amorphous glass or polycrystalline cristobalite form with sub‐200 nm resolution, demonstrating a promising strategy for nanoscale additive manufacturing of silica‐based nanocomposites. Building on this work, TPP was employed to fabricate complex transparent fused silica glass nanostructures, achieving a fourfold resolution enhancement and demonstrating exceptional optical quality, mechanical resilience, and ease of processing.^[^ ^210^ ^]^ Another notable advancement is the photoexcitation‐induced chemical bonding method introduced by Liu et al., which enables additive‐free laser nano printing of semiconductor QD architectures.^[^ ^211^ ^]^ In this method, femtosecond laser pulses generate electron‐hole pairs within the quantum dots, and the photoexcited holes migrate to the nanocrystal surfaces, enhancing their chemical reactivity. The technique achieved a minimum printing linewidth of 81 ± 4 nm and a line edge roughness of 12 ± 2 nm, demonstrating its capability for high‐resolution 3D structuring while preserving the intrinsic optical and electronic properties of the QDs. Additionally, Li et al. introduced a strategy for 3D printing concentrated nanocrystal suspensions without the need for polymer or monomer additives. Their method utilizes nanoparticles naturally capped with ligands, enabling two‐photon irradiation to selectively induce covalent bonding between neighboring particles. This technique makes it possible to directly fabricate micrometer‐scale 3D structures while preserving the original properties of nanocrystals.^[^ ^212^ ^]^ Nanoscale 3D printing continues to face challenges, including low throughput, limited material compatibility, and difficulties in integration with existing device architectures. Although several limitations, this emerging technology is expected to drive fabrication toward higher resolution, greater accuracy, and broader material compatibility. These advances will be critical for enabling next‐generation applications in high‐tech fields such as quantum chip manufacturing, nanophononics, and biomedical nanodevices.

#### 4.2.3 Artificial Intelligence (AI)

Over the past 3 years, AI has emerged as a powerful tool in the assisted fabrication of 3D‐printed functional materials and devices by recognizing patterns, making predictions, and continuously optimizing performance.^[^ ^213^ ^]^ Developing designs specifically for 3D printing technology presents a unique challenge, requiring specialized software and skilled professionals to optimize CAD models for enhanced printer efficiency and functionality.^[^ ^214^ ^]^ AI integration in 3D printing further enhances the design process by providing feature recommendations for existing CAD models, enabling designers to make faster and more informed decisions during the early stages of development.^[^ ^215^ ^]^

In material development, AI accelerates the discovery of suitable resins, bioinks, and metal alloys in 3D printing by analyzing large datasets and predicting optimal formulations. Elbadawi et al. demonstrated this by training conditional generative adversarial networks on a dataset of 1437 FDM‐printed resins, including 336 pharmaceutical‐grade materials in 2024, to optimize design parameters and material properties for improved 3D printing outcomes.^[^ ^216^ ^]^ To validate the predictions, four AI‐generated formulations were successfully fabricated using an FDM printer, highlighting the potential of AI in material innovation and optimization for additive manufacturing.

Additionally, AI algorithms optimize critical printing parameters—such as layer thickness, exposure time, and print speed—to reduce failures and improve resolution. AI's capability to predict the mechanical properties of 3D‐printed materials based on printing parameters is crucial for achieving the desired performance of the final product.^[^ ^217^ ^]^ Yang et al. developed a mathematical model to establish a correlation between the degree of cure and the mechanical properties of materials fabricated via SLA printing.^[^ ^218^ ^]^ This model accurately predicts tensile strength and hardness by integrating multiple parameters, including layer thickness, stratification angle, and curing duration, utilizing an explicit solution algorithm. More importantly, it provides a quantitative assessment of the solidification process for both initial green parts and UV post‐cured components, enabling a high degree of predictive accuracy in determining the mechanical properties of photosensitive liquid resins. The model's predictive capability is demonstrated by accuracy rates of 88% and 90% for tensile strength and 98% and 95% for hardness, for green and UV post‐cured parts, respectively, underscoring its reliability in optimizing SLA‐printed materials.

AI is poised to revolutionize the development of 3D‐printed nanocomposites. Beyond optimizing material formulations, process parameters, and functional properties, AI will play a pivotal role in advancing hybrid nanoparticles and composite inks, driving innovation in additive manufacturing (Figure 9c). Compared to AI‐assisted 3D printing, AI in 3D‐printed nanocomposite research benefits from access to larger and more diverse datasets, including dispersion, concentration, ligand selection, polymer surface grafting, and surface coatings. This extensive data enables the development of more precise and reliable predictive models, enhancing material performance and fabrication efficiency.

#### 4.2.4 Closed‐Loop Recycling in 3D Printing

As the demand for 3D‐printed products continues to rise, concerns over the use of hazardous solvents and the generation of toxic by‐products highlight the urgent need for efficient and sustainable recycling strategies to mitigate environmental impact and material waste.^[^ ^219^ ^]^ One strategy to reduce environmental impact is the development of biodegradable or seawater‐degradable 3D‐printed nanocomposites. However, as these materials degrade, hybrid nanoparticles are released, which often exhibit high stability and resistance to natural decomposition, posing potential environmental problems.

Another promising approach is the development of closed‐loop recycling systems. As shown in Figure 9d, the conceptual recycling process for 3D‐printed nanocomposites consists of three key stages: collection, reprocessing, and reuse. In this approach, discarded nanocomposites are collected and broken down into fine particles through reprocessing techniques such as mechanical grinding, chemical dissolution, or thermal treatment. These processed particles are then repurposed into 3D printing ink, enabling their reintegration into the next printing cycle. This closed‐loop system promotes sustainable additive manufacturing and enhances material circularity. Sevastaki et al. explore the development of nanocomposite polymeric filaments incorporating TiO₂ nanoparticles, derived entirely from recycled solid polystyrene.^[^ ^220^ ^]^ Challenges remain in ensuring recyclability without compromising material integrity, optimizing processing techniques to minimize waste, and evaluating the long‐term performance of recycled nanocomposites. This approach holds promise for 3D‐printed products susceptible to environmental degradation, such as electronic components and heavy metal‐based structures, where sustainable alternatives are increasingly in demand.

## 5 Conclusion

Nanocomposites, which integrate the advantages of both organic and inorganic functional materials, have enabled a wide range of applications across diverse fields. The fabrication of nanocomposites has evolved from traditional synthetic approaches to cutting‐edge 3D printing technologies. This advancement allows for the development of structurally complex and multifunctional materials, expanding their potential in biomedical engineering, soft robotics, electronics, and environmental sustainability.

Despite these advancements, critical challenges persist. The selection and optimization of hybrid nanoparticles must be carefully balanced to ensure uniform dispersion, printability, and compatibility with inks while preserving the structural integrity and functional performance of the final printed constructs. Furthermore, surface engineering techniques require further refinement to enhance the stability of hybrid nanoparticles and promote strong interfacial adhesion within the composite matrix.

Emerging technologies, including AI, nanoscale additive manufacturing, and 5D printing, are poised to drive the next phase of innovation in 3D‐printed nanocomposites. AI, in particular, offers transformative potential by accelerating material discovery, optimizing printing parameters, and enabling predictive modeling for enhanced performance customization. Meanwhile, advances in high‐resolution printing techniques are expected to facilitate the precise fabrication of nanocomposites at the nanoscale, further expanding their functional applications in next‐generation biomedical devices, flexible electronics, and energy storage systems. By addressing these challenges and leveraging emerging technologies, 3D‐printed nanocomposites have the potential to revolutionize multiple industries, contributing to the development of smarter, more adaptive, and environmentally responsible materials.

## Conflict of Interest

The authors declare no conflict of interest.

## Author Contributions

L.Z. and X.H. contributed equally to this work. R.Q. and L.W. conceptualized the review. L.Z. contributed to data collection, outlined the structure, and led the manuscript writing. X.H., L.L., and N.K.A.N. contributed to the literature review and drafting of specific sections. X.Z, T.P.D., and L.W. contributed to the revision. R.Q. supervised the project, reviewed the manuscript, and provided critical feedback for improvements.
